# Perforator-Aware Explainable Microsurgical Intelligence for Basilar Trunk Aneurysm Reconstruction: A Human-Centred Clinical AI Framework for Precision Neurovascular Surgery—A Retrospective Single-Centre Study

**DOI:** 10.3390/jcm15166147

**Published:** 2026-08-07

**Authors:** Matei Șerban, Corneliu Toader, Alexandru Vlad Ciurea, Leon Dănăilă, Răzvan-Adrian Covache-Busuioc

**Affiliations:** 1 Department of Neurosurgery, “Carol Davila” University of Medicine and Pharmacy, 050474 Bucharest, Romania; mateiserban@innbn.com (M.Ș.);; 2 Department of Vascular Neurosurgery, National Institute of Neurology and Neurovascular Diseases, 077160 Bucharest, Romania; 3 Puls Med Association, 051885 Bucharest, Romania; 4 Medical Section, Romanian Academy, 010071 Bucharest, Romania

**Keywords:** basilar trunk aneurysm, microsurgery, explainable artificial intelligence, perforator injury, pontine infarction, brainstem compression, clip reconstruction, hidden disability, surgical decision support

## Abstract

Basilar trunk aneurysms (BTAs) are rare intracranial aneurysms. These aneurysms pose substantial clinical risk because they are located close to vital cranial structures and perfusion-sensitive perforating arteries. In addition, outcomes from BTA repair are dependent upon several factors, which include proximity to the brainstem, preservation of perforators, corridor access to the aneurysm, successful clipping of the aneurysm through reconstruction, the need for intraoperative rescue manoeuvres and the ability of the patient to recover from complications. Many previous studies have documented the outcomes associated with BTA repair; however, few studies have examined how the anatomy of the BTA directly relates to surgical decisions made by surgeons. Therefore, we developed an explainable AI framework for documenting surgeon reasoning regarding the open microsurgical treatment of BTAs. The primary objective was to develop and internally evaluate an explainable microsurgical intelligence framework for structuring surgeon reasoning during open microsurgical treatment of BTAs. The secondary objectives were to explore the relationships between the proposed constructs and postoperative pontine infarction, angiographic occlusion, functional outcome, hidden disability, operative difficulty, and composite technical-safety failure. **Methods:** We retrospectively analysed the cases of 31 adult patients who underwent open microsurgical treatment of a basilar trunk aneurysm at our hospital between October 1999 and March 2025. The cohort included 18 women (58.1%) and 13 men (41.9%), with a median age of 55 years (interquartile range, 44–63 years); 14 patients (45.2%) presented with ruptured aneurysms. A database containing more than 300 variables collected information about each patient’s imaging studies, operative strategies employed during surgery, intraoperative events encountered during surgery, intraoperative angiographic verification, occurrence of new injuries or complications resulting from surgery, and degree of recovery in each patient. Temporally separated scores were generated to quantify perforator-aware hazard, compression burden imposed by proximity to the brainstem, constraints imposed by corridors available for clipping of the BTA, burden imposed by clip reconstruction, degree of surgical precision adjusted based on the need for rescue manoeuvres, degree of dataset/model readiness, and degree of case-level learning density. The framework was evaluated internally using methods that included leave-one-out cross-validation, Firth regression modelling, Bayesian modelling, bootstrap optimism correction, calibration assessments using Brier score, decision-curve analysis, evaluations of explainability, conformal uncertainty estimation, and retrieval of similar cases. **Results:** Complete occlusion of the aneurysm was successfully achieved in 27 patients (87.1%), whereas residual neck or sac remained in four patients (12.9%). New pontine infarction occurred in six patients (19.4%), including four perforator-related infarctions (12.9%). A favourable last-follow-up modified Rankin Scale (mRS) score of 0–2 was achieved in 25 patients (80.6%); however, hidden disability was noted in 11 of these 25 patients (44.0%). Mortality was 6.5% and was limited to two patients with high-grade rupture. PAH-S-pre predicted pontine infarction (OR, 1.19 for every five-point increase; leave-one-out cross-validated AUC, 0.92). The composite technical-safety failure model had an ROC AUC of 0.91, optimism-corrected AUC of 0.88, calibration slope of 0.96, Brier score of 0.10, and permutation *p* < 0.001. Explainability indicated that PAH-S-pre, BPCI, and CCR represented the most important features. Conformal prediction resulted in abstention from prediction in four patients (12.9%). **Conclusions:** Expert microsurgical thought processes involved in repairing BTAs can be systematised and recorded into time-relevant and clinically meaningful measures that preserve anatomical interpretability. Clinical use will require external validation before implementation; however, this framework may provide a clinically interpretable foundation for risk-adapted planning, verification, surveillance, education, and future decision-support research for complex neurovascular surgery.

## 1. Introduction

Basilar trunk aneurysms are a rare and especially difficult-to-treat group of intracranial aneurysms [1] that have the unique characteristic of multiple critical structures—the aneurysm wall, brainstem perforating vessels, branches of the anterior inferior cerebellar artery and superior cerebellar artery, cranial nerves, the parent vessel, and the ventral surface of the brainstem—being located within millimetres of each other [2,3], making even slight clip malposition, suboptimal visualisation, or decreased flow through perforators potentially disastrous because of brainstem injury [4]. Therefore, treatment success depends not only on aneurysm size, rupture status, occlusion status, mortality, and modified Rankin Scale outcomes, but also on accurate anatomical understanding, surgical access and exposure, parent-vessel repair or reconstruction, perforator preservation, and timely identification of reversible hazards [5].

Although endovascular therapy has improved significantly over time, open clipping and reconstructive microsurgery continue to be applicable in selected cases involving complex morphology, thrombus or calcification, major branch incorporation, fusiform or dissecting pathology, brainstem mass effect, previous failed treatment, or the need for durable repair or reconstruction [6].

However, the low incidence, anatomical heterogeneity, small sample sizes available for basilar trunk aneurysm studies, and potential for severe treatment-related morbidity and mortality all contribute to the difficulty of studying these lesions using traditional statistical methods [7,8,9].

Most current databases report events occurring during treatment, such as rupture, occlusion, infarction, disability, retreatment, or death [10,11]; however, fewer provide information regarding why a particular case was challenging, whether hazards were recognised before treatment, and how these hazards were addressed or managed. Cases involving aneurysms of similar size and comparable patient outcomes can exhibit significant differences in terms of perforator involvement, degree of brainstem compression, corridor availability and accessibility, direction and trajectory of clip placement, reconstruction burden, verification findings, and the requirement for rescue techniques [12,13].

Increasingly, artificial intelligence and machine learning are being used in neurosurgery for diagnosis, planning, and outcome prediction. However, most existing AI-based models primarily use routinely collected clinical and imaging data. Additionally, issues related to interpretability, external validation, and clinical transportability remain limitations of these applications [14,15].

Therefore, our framework focused on developing a method to represent microsurgically derived information about anatomy, exposure, reconstruction, verification, rescue, injury, and recovery in a structured manner. To accomplish this, we developed temporally separated and anatomically interpretable metrics relating to perforator-related hazard, brainstem vulnerability, corridor constraints, reconstruction burden, rescue-adjusted performance, dataset readiness, model interpretability, and case-level learning density. Our primary objective was to develop and internally test this explainable microsurgical intelligence (EMI) framework in patients undergoing open treatment for basilar trunk aneurysms. Our secondary objectives were to examine associations with postoperative pontine infarction, complete occlusion at follow-up, postoperative hidden disability, operative difficulty, and composite technical-safety failure. The EMI framework was intended to serve as an auditing tool and research structure rather than a stand-alone clinical decision-support application.

## 2. Materials and Methods

### 2.1. Study Design and Dataset Description

#### 2.1.1. Study Background

In this retrospective, single-centre, proof-of-concept study, we developed and internally evaluated an explainable microsurgical intelligence (MSI) framework to assess the applicability of this framework for the evaluation of basilar trunk aneurysms treated by either open microsurgical clipping or open reconstructive microsurgery. Due to the low incidence of these aneurysms, few cases are reported in the literature, and there is limited information about how best to evaluate them based on their anatomical features. Thus, our study was conducted within the context of rare, high-risk cerebrovascular surgery, where expert surgeons have historically made complex decisions using expert evaluations of aneurysm anatomy, perforator risk, proximity to the brainstem, corridor geometry, clip reconstruction, intraoperative verification, and potential rescue manoeuvres.

Our main methodological goal was to convert expert microsurgical reasoning into a set of structured, temporally ordered, clinically interpretable variables that would enable anatomical risk assessment, technical-performance evaluation, and outcome modelling, and provide a foundation for future studies of decision-support tools. Our study was not intended to serve as a definitive clinical trial evaluating the safety and efficacy of our MSI framework before any prospective clinical use. Therefore, all predictions generated from our MSI framework should be viewed as internal feasibility indicators requiring further evaluation outside our institution. No MSI output influenced clinical decision-making or patient management. Although the study was retrospective, preoperative and intraoperative predictor scores were assigned from temporally restricted, outcome-masked source packages and were finalised before linkage to postoperative imaging, angiographic, and clinical endpoints.

The reporting of this study was guided by the Transparent Reporting of a Multivariable Prediction Model for Individual Prognosis or Diagnosis Plus Artificial Intelligence (TRIPOD+AI) statement for studies that develop or evaluate clinical prediction models using regression or machine-learning methods [16]. Applicable reporting recommendations were incorporated throughout the Methods, Results, and Discussion sections, including descriptions of the data sources, participant selection, outcomes and predictors, missing-data handling, model development, internal validation, performance assessment, and study limitations.

#### 2.1.2. Study Population

We identified all patients who underwent open microsurgical clipping or reconstructive microsurgery for basilar trunk aneurysms at the National Institute of Neurology and Neurovascular Diseases in Bucharest, Romania, from October 1999 through March 2025. Prior to initiating this study, we obtained approval from the Institutional Review Board of the National Institute of Neurology and Neurovascular Diseases in Bucharest, Romania (No. 6877, approved on 23 July 2025), which waived informed consent for anonymised retrospective data. All patient data were anonymised and handled in accordance with institutional privacy guidelines and applicable Romanian and European Union laws governing data protection, including GDPR requirements.

#### 2.1.3. Definition of Basilar Trunk Aneurysms

For the purposes of this study, a basilar trunk aneurysm was defined as one originating from the basilar artery segment between the vertebrobasilar junction and the origin of the superior cerebellar arteries. Pure basilar apex aneurysms and pure vertebrobasilar junction aneurysms were excluded from consideration to maintain anatomical specificity.

#### 2.1.4. Selection Criteria

Patients were selected for inclusion if they met the following criteria: (1) age ≥18 years; (2) aneurysm confirmation by digital subtraction angiography (DSA), CT angiography, 3D rotational angiography, magnetic resonance angiography (MRA), or multimodal vascular imaging; (3) treatment by open microsurgical clipping, clip reconstruction, trapping, bypass-assisted clipping, or another form of open reconstructive microsurgery; and (4) availability of a standardised core dataset spanning the preoperative, intraoperative, postoperative, and follow-up periods. Patients were excluded if: (1) their aneurysm originated outside the specified basilar trunk region; (2) they had previously undergone treatment that rendered reconstruction of the original open surgical anatomy impossible; (3) the procedure lacked an open reconstructive component; (4) they had traumatic brain injury; (5) they had an intracranial neoplasm; (6) they had infectious arteriopathy; or (7) they had another major neurological disorder that could complicate outcome attribution.

#### 2.1.5. Summary of Patient Cohort

The analytical cohort included 31 consecutive adult patients. Median patient age was 55 years (interquartile range [IQR], 44–63). Median body mass index (BMI) was 27.4 kg/m^2^ (IQR, 24.1–31.2). A total of 7 patients (22.6%) were classified as obese. Fifteen patients (48.4%) were classified as American Society of Anesthesiologists Physical Status class ASA I–II; 16 patients (51.6%) were classified as ASA III–IV. Comorbidities seen most frequently included arterial hypertension in 19 patients (61.3%), dyslipidaemia in 14 (45.2%), diabetes mellitus in 5 (16.1%), ischaemic heart disease in 4 (12.9%), and previous stroke or transient ischaemic attack in 3 (9.7%). In addition to their comorbidities, 10 patients (32.3%) were current or former cigarette smokers. Thirteen patients (41.9%) experienced neurological deficits prior to undergoing surgery; 9 (29.0%) underwent surgical intervention for symptomatic mass effect or cranial nerve involvement. The demographic and clinical presentation characteristics of this population are listed in Table 1.

#### 2.1.6. Collection and Evaluation of Data

Clinical data were collected from electronic medical records, operative reports, anaesthesia records, intensive care unit documents, postoperative monitoring charts, discharge summaries, outpatient follow-up notes, institutional imaging databases, postoperative computed tomography scans, MRI studies with diffusion-weighted imaging sequences, angiographic follow-up examinations, and intraoperative video recordings when available. Clinical data were abstracted by a multidisciplinary group consisting of vascular neurosurgeons and neuroradiologists. Any disagreements regarding abstraction were resolved via consensus among members of this group. Priority was placed on accurate determination of aneurysm localisation, perforator involvement, need for intraoperative rescue, presence of postoperative diffusion-weighted imaging-defined injury, final angiographic occlusion status, and attribution of neurological outcomes to specific treatments.

#### 2.1.7. Blinding, Temporal Masking and Independent Score Assignment

The overall design for the longitudinal database was developed prior to the development of the predictive scores (i.e., the model) that were used to predict postoperative outcomes. In order to reduce the potential for outcome-informed rater bias, both preoperative and intraoperative rating scores were determined using temporally limited source packages, and all raters involved in these assessments were blinded to postoperative imaging and clinical outcomes.

All preoperative ratings (e.g., PAH-S-pre, BPCI, etc.) were based solely on clinical and vascular imaging data that were available prior to surgical incision. Raters making these assessments had access to none of the following data: operative reports, intraoperative video recordings, intraoperative verification findings, postoperative CT scans, diffusion-weighted MRI findings, postoperative angiographic occlusion status, discharge neurological status, modified Rankin Scale (mRS) scores, subsequent treatment, mortality, or long-term follow-up. All preoperative ratings were completed and finalised before being linked to the respective postoperative outcomes.

Subsequent intraoperative ratings (e.g., PAH-S-intra, RASP-execution, etc.) were made using operative data available at the time of completion of surgery and therefore prior to obtaining postoperative imaging. The raters had access to the operative report, intraoperative videos when available, clip deployment and repositioning, intraoperative verification findings, neuromonitoring data, and rescue manoeuvres employed. However, they remained masked to postoperative DWI findings, postoperative angiographic findings, discharge outcome, mortality, and long-term follow-up. Similar to the preoperative ratings, all intraoperative ratings were also finalised before postoperative outcomes were disclosed.

Postoperative imaging findings, angiographic occlusion status, functional outcome measures (including mRS), and mortality were assessed independently of both preoperative and intraoperative ratings. PAH-S-post, CRB-final, and RASP-final were created solely during this postoperative adjudication process and were never entered as predictors of outcomes incorporated within their respective definitions.

Each of the two independent raters completed the initial rating assignments without knowledge of the other rater’s initial ratings. When disagreements arose regarding the same case, the raters were not allowed to discuss or influence one another until after the individual ratings had been submitted. Disagreements were then resolved through consensus between the two raters. If necessary, a third senior neurosurgeon was available to act as an arbiter and assist in reaching consensus. Of the 10 reliability cases examined, none required third-party arbitration. This design separated the temporal eligibility of each variable considered in the model from outcome masking and ensured that neither the preoperative nor the pre-outcome intraoperative rating assignments were influenced by knowledge of postoperative endpoint values.

#### 2.1.8. Registry Structure

Our registry was organised as a high-resolution microsurgical intelligence database rather than an administrative database. The database contains more than 300 variables grouped into four layers: (1) anatomical-imaging layer; (2) operative-decision layer; (3) postoperative injury and angiographic layer; and (4) longitudinal functional-recovery layer.

Variables within the anatomical-imaging layer are based upon the location, size, orientation, morphology, and shape of the basilar artery segments; neck and dome morphometric measurements, including diameter, angle, and length; the location and direction of the neck and its origin; the relationship of the posterior circulation to the basilar artery; geometric spatial relationships between the vertebral arteries and the basilar artery; branching patterns of cerebral blood vessels and variations in these patterns; anatomical factors derived from development, including embryological configurations or fenestrations within the cerebral vasculature; and all other anatomical factors pertinent to the case.

Preoperative perforator variables included the total number of perforators that could be identified through visualisation or suspected on vascular imaging; the proximity of each perforator to either the aneurysm neck or sac; whether imaging suggested that one or more perforators originated from the neck or sac; whether multiple perforators appeared to be clustered together; whether it seemed likely that one or more perforators were incorporated into the aneurysm neck or sac; whether one or more perforators were believed to be concealed from view behind the aneurysm dome, neck, or an immediately adjacent branch-bearing surface; the potential for one or more perforators to enter the surgical field or conflict with clip placement; territories potentially at risk; the overall pontine perforator density; and confidence in the assessment made from the imaging studies used.

Intraoperatively confirmed variables included adherence, perforators concealed from view by the operative exposure used to access the aneurysm or obscured by the clip construct placed across the aneurysm neck, direct observation of a clip line crossing a perforating vessel, difficulty encountered during dissection and access to the aneurysm, and intraoperative flow disturbances. These items were classified as belonging exclusively to the operative layer because they became identifiable only after surgery had commenced.

Brainstem- and cranial nerve-related variables included the distance from the ventral pons to the aneurysm dome or neck; the degree of brainstem contact; compression grade or volume; pontine oedema; midbrain or fourth ventricle compression; hydrocephalus due to mass effect; cranial nerve contact grades; neurovascular conflict symptoms; and pre-existing deficits involving cranial nerves III–XII.

Variables in the operative layer were designed to capture both the temporal order and sequence of the surgeon’s decision-making process rather than simply the final angiographic appearance. These variables include indications for open surgery, surgical approach, craniotomy side, skull base extensions, primary and secondary corridors, proximal and distal control, temporary clipping, adenosine or rapid pacing, bypass or revascularisation planning, parent-vessel preservation, trapping, wrapping, indocyanine green angiography, micro-Doppler, endoscopic assistance, intraoperative DSA, and neuromonitoring. Additional operative variables included achieved working distances, widths, and depths; microscope and clip-applier angles; direct visibility of the neck, dome, parent artery, perforators, and proximal and distal basilar artery; operative blind spots; directly confirmed adherence or adhesions; perforators concealed by the exposure or clip construct; and observed conflict between perforators, branches, and the applied clip line. Clip reconstruction variables include clip count, clip type, fenestrated or non-fenestrated clips, angled, curved, straight, or mini-clip usage, pilot clip usage, tandem, stacked, parallel, reconstructive, or parent-vessel-preserving constructs, booster clips, remodelling of the dome or neck, clip slippage, repositioning, and final construct configuration.

Each operation was assessed as a series of sequential decisions related to: (1) indication for open reconstruction; (2) approach choice, craniotomy side, and skull base extensions; (3) corridor geometry; (4) proximal and distal control; (5) perforator exposure and visualisation; (6) clip reconstruction strategy; (7) intraoperative verification; (8) rescue manoeuvres; and (9) postoperative injury assessment. We focused particular attention on whether the operative plan accounted for the predominant anatomical hazards encountered during the procedure, whether adequate visualisation occurred before permanent clipping was applied, whether temporary occlusion was minimised or required because of reconstructive needs, and whether any changes in intraoperative findings prompted corrective actions before closure.

#### 2.1.9. Postoperative and Follow-Up Variables

Additional variables included postoperative imaging status, angiographic occlusion status, residual neck or sac, parent basilar artery stenosis, branch or perforator compromise, diffusion-weighted imaging lesions, pontine and non-pontine infarcts, perforator-territory infarct adjudication, ICU and hospital course, discharge neurological status, discharge modified Rankin Scale score, Glasgow Outcome Scale score, mortality, longitudinal modified Rankin Scale score, longitudinal Glasgow Outcome Scale score, durability, recurrence, growth, clip migration, stenosis, retreatment, cognitive and functional recovery, work status, and quality-of-life data whenever available. Hidden disability was defined as any persistent cognitive, fatigue-related, gait, balance, swallowing, mood, behavioural, return-to-work, or quality-of-life impairment despite generally favourable functional outcome, conventionally indicated by mRS 0–2.

#### 2.1.10. Complexity and Video Assessment

Structured complexity and video-review variables included BTOCC component and total scores, video-record availability, video quality, exposure quality, anatomical structures visible during video review, perforator dissection, temporary clipping, clip application, indocyanine green angiography, micro-Doppler, intraoperative rupture, clip repositioning, final construct formation, video completeness, video-based perforator grade, video-based clip-complexity grade, and video-based corridor grade. These variables allowed individual case histories to function as structured units of learning linking anatomical considerations with operative execution, intraoperative verification, rescue, imaging, outcome, and durability.

### 2.2. Data Preprocessing

The purpose of the preprocessing was to ensure that temporal eligibility was preserved, that outcome masking was maintained, and that the actual microsurgical significance of each variable was preserved by eliminating direct predictor–outcome leakage. Each variable was categorized into a total of seven different time layers (preoperative clinical and radiologic data; planned surgical strategy; intraoperative exposure and execution; intraoperative verification; postoperative radiologic findings; post-discharge status; and long-term follow-up).

All models to be employed prior to making an initial skin incision utilized only data available at the time of decision-making, with all relevant scores being generated from outcome-masked preoperative data sources. All models representing intraoperative updates contained both operative and verification-related data that were available immediately prior to postoperative radiologic evaluation, and in all cases, raters remained blinded to both postoperative imaging results and clinical outcomes. All postoperative data, including imaging findings, angiographic occlusion, post-discharge status, mortality rates, and long-term patient outcomes, were evaluated as endpoints, adjudicated variables, or quality metrics and/or were otherwise not permitted to serve as predictors of the aforementioned outcomes.

Values that were structurally missing based on clinical relevance were categorised accordingly. Values that were structurally missing due to lack of applicability, such as Hunt–Hess grade, WFNS grade, Fisher grade, clot distribution, or time from rupture to surgery in unruptured patients, were encoded as structurally missing and not imputed. Values missing due to lack of documentation were coded as not recorded and were interpreted in relation to treatment era, imaging availability, documentation density, or video availability. Missing values related to documented assessments, such as video-based grading in documented video cases, were coded as separate states. Low-frequency missing values related to predictor variables were imputed only if clinically relevant and never where they would indicate a complication. No postoperative infarct, occlusion result, mortality, modified Rankin Scale, Glasgow Outcome Scale, need for additional treatment, or hidden disability outcome was ever imputed. Models used values from the same or previous temporal layers only for imputation purposes. Predictive mean matching was used to impute continuous predictors; anatomically stratified median imputation was used to impute selected continuous predictors when appropriate; logistic regression was used to impute binary predictors; proportional-odds regression was used to impute ordinal predictors; and polytomous regression was used to impute nominal predictor variables. Use of random forest imputation was limited to sensitivity analyses.

Continuous variables were reviewed for internal consistency of units, biological plausibility, outliers, and overall structure. When internally valid and biologically plausible, these variables were evaluated continuously. Otherwise, continuous variables were converted into discrete categories only when this conversion provided clear clinical interpretation relative to their underlying biology. Maximum aneurysm diameter was categorised into four groups: <7 mm, 7–12 mm, 12–20 mm, and >20 mm. Neck width was categorised into four groups: <4 mm, 4–6 mm, 6–10 mm, and >10 mm. Temporary occlusion time was categorised into five groups: 0 s, <180 s, 180–300 s, 300–600 s, and >600 s. Perforator burden was represented through subgroups including counts of perforators within 3 mm and 5 mm of the neck, incorporation of perforators, sac-origin perforators, and perforator clusters. Corridor difficulty was represented by both raw measurements and geometric ratios. Ordinal variables, including perforator incorporation, perforator density, perforator confidence scores, visibility, brainstem compression, cranial nerve contact, quality of control, BTOCC class, modified Rankin Scale, and Glasgow Outcome Scale, were retained as ordered variables. Circular-distance encoding and sine-cosine encoding were applied to clock-face variables.

Feature engineering served as the principal method for reducing dimensionality in this study. Rather than forcing hundreds of raw variables into large-scale models that may have had many parameters to estimate, feature engineering was used to develop several composite variable scores that corresponded to specific aspects of microsurgery. All scores had a predefined direction that was intended to capture unique aspects of each aneurysmal construct; all scores also had predefined timing that helped define what temporal layers should be utilised when developing predictive models. Finally, each score had a predetermined analytic use that reduced temporal ambiguity and prevented the phenomenon known as outcome leakage. Unless a threshold for clinical interpretation was previously established by clinicians in a manner that implied the threshold was a prespecified anchor point for exploration, these thresholds are referred to hereafter as exploratory anchor points. The architecture for constructing these score types is summarised in Table 2.

The SAPIENS architecture was composed of three main components: SAPIENS-Dataset, SAPIENS-Model, and BTA-SAPIENS-Case. SAPIENS-Dataset is a construct designed to evaluate how well a particular dataset can serve as a registry-readiness construct; this construct is summarised across all contributing cases by using case-level contribution scores. SAPIENS-Dataset was not directly used as a clinical predictor in this study.

SAPIENS-Model represents the degree of success achieved by each model type in terms of discrimination, calibration, interpretability, clinical utility, uncertainty, and human oversight. Discrimination refers to how well the model distinguishes between different levels of risk; calibration refers to whether the model assigns higher predicted risks to individuals who actually experience adverse events; interpretability refers to how understandable the output of the model is; clinical utility refers to whether the model is useful for guiding decisions made by clinicians; uncertainty refers to how uncertain the output of the model is; and human oversight refers to whether expert oversight was involved in generating or interpreting the predictions made by the model.

Finally, BTA-SAPIENS-Case represents how adequately each aneurysm was characterised so that it can function as a single structured learning unit.

The RASP score was developed in two parts: RASP-execution and RASP-final. RASP-execution utilised only intraoperative variables available before postoperative imaging. However, RASP-final also included penalties related to postoperative harm. These included pontine infarct, residual sac, need for retreatment, or death attributable to surgical complications; therefore, RASP-final was analysed as a technical-performance endpoint rather than being considered an independent predictor of postoperative injury.

The PAH-S model is a three-part system. There are PAH-S-pre, PAH-S-intra, and PAH-S-post layers within the PAH-S architecture. The PAH-S-pre layer includes all information regarding anatomical detail and clinical data, in addition to relevant imaging obtained prior to the surgical procedure and/or reasonably expected to be identifiable before the surgeon made the initial incision. In contrast, the PAH-S-pre layer does not include directly identified adherence, perforating vessels obscured by either the operative exposure or the placement of clips, observed relationships between perforators and clip lines, difficulties encountered with dissection during surgery, verification findings, or rescue manoeuvres undertaken as part of the repair.

In addition to the PAH-S-pre layer, there is another layer referred to as PAH-S-intra. The PAH-S-intra layer incorporates information related to the operative procedure that became available subsequent to surgical exposure and prior to obtaining postoperative imaging. Included within this layer of assessment are direct visualisation of anatomical structures, confirmation of adherent tissue, visualisation of the relationship between perforators and clip lines, suspicion of compromised integrity, verification findings, and corrective actions undertaken as part of the repair. Raters assessing PAH-S-intra remained blinded to postoperative diffusion-weighted imaging-defined injury, postoperative angiographically defined occlusion status, postoperative mortality, and functional outcome. The PAH-S-intra layer therefore represents an intraoperative pre-outcome updating layer rather than a postoperative adjudication layer.

Finally, there is a third layer called PAH-S-post. This layer can only be utilised for postoperative mechanistic adjudication and cannot be used to predict whether an ischaemic lesion occurred based upon diffusion-weighted imaging criteria. As such, the overall structure provides a means of both separating the timing of assessments and masking outcomes from those who assign scores; preoperative and intraoperative scores were finalised prior to disclosure of postoperative outcomes, while all outcome-based variables were relegated to the postoperative adjudication layer.

The Brainstem Proximity-Compression Index combines brainstem proximity, contact, compression, oedema, mass effect, hydrocephalus, and cranial nerve contact burden.

The corridor constraint ratio is defined as the ratio of aneurysm depth to working corridor width.

Clip reconstruction burden captures reconstructive technical burden prior to outcome incorporation. CRB-predictor excludes residual neck or sac; CRB-final includes final residual morphology descriptively.

BTOCC total and component scores provide a reference microsurgical complexity framework against which derived complexity constructs can be compared.

Collinear derived scores were examined via correlation matrices and variance-inflation diagnostics when applicable. Derived scores with overlapping content were not simultaneously entered into small-sample models unless clearly justified based on clinical context. Priority in selecting derived scores for inclusion was placed upon anatomical interpretability of the derived score, temporal validity of the derived score, penalisation, stability across resampling procedures, and clinical plausibility.

### 2.3. Data Splitting and Internal Validation Strategy

The cohort of 31 patients was too small for a random 80/20 split into training and validation groups. Therefore, the whole cohort was used for final model development. To ensure that sufficient data were used during internal validation while still providing an internal estimate of performance in unseen cases, rare-disease resampling techniques were employed. Leave-one-out cross-validation (LOOCV) was used as the primary method of internal validation. In this procedure, each patient was removed from the cohort once, and the model was trained using the remaining 30 patients. The fitted model was then applied to the held-out patient. This process generated one strictly out-of-fold prediction for each of the 31 patients.

Each LOOCV iteration began before any data-dependent preprocessing or model estimation. Imputation, scaling, feature selection, threshold derivation, hyperparameter selection, calibration, and model fitting were performed using only the 30-patient training set. The held-out patient was excluded from all data-dependent procedures until the final prediction was generated. Score definitions and weights were fixed independently before outcome analysis and were therefore not re-estimated within the cross-validation iterations.

The resulting out-of-fold predictions were used to calculate measures of discrimination, calibration, classification behaviour, decision-curve net benefit, explanation stability, and uncertainty. Although LOOCV requires repeated model refitting, it maximises the amount of data available for model training at each iteration while still generating out-of-fold predictions. Given the small cohort and sparse endpoint structure, the resulting performance estimates were interpreted as exploratory internal estimates rather than as evidence of external validation.

Hyperparameters are usually chosen based on their impact on model performance. However, if there are many hyperparameters, it may become difficult to find a combination of hyperparameters that results in optimal performance, and extensive tuning may increase the risk of overfitting in small datasets. This problem can be addressed by employing an optimisation algorithm to find a combination of hyperparameters that minimises the error function of the model. One such algorithm is grid search. Grid search works by defining a set of values for each hyperparameter and then running the model multiple times with different combinations of these values. This leads to a trade-off between computational cost and potentially finding better combinations of hyperparameters. In this study, when hyperparameter tuning was required for exploratory machine-learning models, the search space was kept limited and prespecified.

Alternatively, algorithms that do not rely on brute-force searches can be employed. Examples include gradient-based optimisation, genetic algorithms, simulated annealing, and particle swarm optimisation. All of these algorithms may work well in certain situations but require specific parameterisation depending on the problem they need to solve. Given the small cohort size and sparse endpoints in this study, these approaches were not used as primary model-selection strategies.

Additionally, some researchers employ Bayesian optimisation. Bayesian optimisation also relies on the concept of searching for better solutions over a predefined area of possible solutions. However, unlike deterministic methods such as grid search, Bayesian optimisation employs probability theory to reduce the number of evaluations required to reach a solution. In this study, Bayesian optimisation was not used as a primary tuning strategy, because the priority was placed on interpretability, stability, clinical plausibility, and avoidance of overly optimised solutions in a rare-event dataset.

Bootstrap resampling was also used to quantify optimism, defined as the difference between apparent and resampled performance, as well as uncertainty and model stability. If enough data existed to allow model refitting after bootstrapping, up to 2000 bootstrap replicates were created for prespecified primary models that had sufficient endpoint structure. Models were refitted on each replicate, and apparent versus resampled performance was compared to estimate optimism. Confidence intervals were constructed for AUC, PR-AUC, Brier score, calibration slope, calibration intercept, MCC, regression coefficients, feature importance, explanation stability, and decision-curve net benefit using non-parametric bootstrap methods.

Permutation testing was performed by randomly permuting the outcomes while keeping the predictor matrix unchanged. Observed performance metrics could then be compared against those expected due to randomness alone.

Treatment era was analysed as a secondary dimension of interest to study how surgical practice evolved over time for various technologies and innovations that occurred during the time spanned by the cohort, from 1999 to 2025. Technologies and innovations studied included 3D angiography, vessel-wall MRI, neuronavigation, indocyanine-green angiography, micro-Doppler, intraoperative DSA, neuromonitoring, availability of operative videos postoperatively, postoperative DWI scans, clip technology, and documentation density.

Internal validation assessed whether the proposed scores conveyed greater than chance expectations, whether estimated performance metrics demonstrated stability under resampling conditions, whether explanations remained relevant to surgical decisions, and whether uncertainty was able to identify cases beyond the range of reliable experience documented in the registry.

### 2.4. Algorithm Description

Layered modelling strategies were implemented for developing models from rare-event interpretable statistics through constrained nonlinear exploratory models and implementation-oriented decision-support systems. Conservative primary models were developed with emphasis placed upon interpretability relative to clinicians’ needs. Nonlinear exploratory models were developed to evaluate nonlinear relationships present in the data and translate them into readable formats for surgeons. Modules were designed to demonstrate potential support for surgeons’ reasoning processes during surgery, such as intraoperative rescue analysis, postoperative surveillance activities, and quality-monitoring activities; however, none were considered ready for deployment as clinical tools.

Firth penalised logistic regression was employed as the base model for binary-outcome problems, since relatively small sample sizes and sparse clinical endpoints often lead to separation in maximum-likelihood estimates, which can provide biased estimates. Model development was completed using candidate predictor variables limited to clinician-derived variables: PAH-S-pre, PAH-S-intra, BPCI, CCR, CRB-predictor, BTOCC total or class, rupture status, anatomical diameter, neck width, temporary occlusion time, and RASP-execution. Odds ratios (ORs), profile penalised-likelihood confidence intervals (PPLCIs), penalised log-likelihood ratio tests (PLLRTs), and predicted probabilities (PPs) were reported. Bayesian logistic regression was employed as an auxiliary model to Firth logistic regression to provide uncertainty assessments through posterior distributions of parameters. Weakly informative priors were employed so that clinical effect sizes could be preserved while providing stabilised estimates. Posterior summaries included median ORs, 95% credible intervals for ORs, posterior probabilities of clinically meaningful effects, and posterior predictive checks (PPCs).

Bayesian cumulative logit models were employed for estimating discharge modified Rankin Scale (mRS), mRS at 3-month follow-up, mRS at 6-month follow-up, mRS at last follow-up, and Glasgow Outcome Scale (GOS) at last follow-up while preserving the ordinal structure of outcomes.

Penalised regression models were only applied to reduced predictor sets consisting of derived scores along with prespecified essential covariates. Ridge regression was employed for correlated constructs representing anatomical risk; LASSO was employed for selective feature extraction through exploratory modelling; and elastic net was employed for selective feature extraction while accounting for grouped correlation between predictors. Selection frequency across bootstrap samples was used to discriminate between stable signals and small-sample artefacts.

Exploratory machine-learning models included shallow classification and regression trees (CART), random forest (RF), extreme gradient boosting (XGBoost), and explainable boosting machines (EBM). These models were viewed as nonlinear benchmarks rather than definitive clinical prediction tools. CART models were restricted to depths of 2–3 so that readable rules could be generated using the output from these models. RF was employed as a constrained nonlinear comparator against which performance metrics could be compared. XGBoost was employed with a low learning rate, limited depth, and regularisation constraints to prevent overfitting. EBMs provided nonlinear but interpretable risk functions under monotonic constraints: predicted risk was not allowed to decrease with increasing values of PAH-S-intra, BPCI, CCR, or temporary occlusion time; nor was predicted risk allowed to increase with improved values of RASP-execution unless such improvement was clinically plausible according to a prespecified interaction.

The surgical digital twin module was developed as a weighted case-similarity retrieval system rather than as a generative simulation. Each case was represented by planning-level and intraoperative features, including basilar trunk segment identified during surgery; clock-face orientation and surface projection of the neck region identifiable during surgery; projection axis determined during surgery; morphological characteristics of the aneurysm and brainstem compression; planning approach selected for the surgical procedure; planned control strategy; planned clip reconstruction; PAH-S-pre; BPCI; CCR; brainstem and cranial nerve engagement; age; rupture status; anatomical diameter; neck width; temporary occlusion time; and RASP-execution. Circular variables were represented using circular distance, while categorical variables were represented using weighted matching. Weights assigned to perforator and corridor features were highest, those assigned to morphology and brainstem proximity were intermediate, and those assigned to demographic features were lowest. For each index case, the three most similar prior cases were retrieved based on these weights, together with their surgical approaches, anatomical hazards, clipping strategies, verification steps, rescue actions, diffusion-weighted imaging findings, and functional outcomes.

This module was designed as an exploratory case-based learning architecture and potential planning-support tool requiring external validation before use in a clinical setting.

Verification activities completed intraoperatively were analysed as diagnostic and rescue systems. Methods used for visualisation and detection of residual blood flow, including indocyanine-green angiography, micro-Doppler techniques for patency of branches and the basilar artery, endoscopic assistance, intraoperative DSA, and neuromonitoring, were compared against postoperative angiography results, postoperative imaging findings, and clinical outcome measures documented during follow-up.

Indocyanine-green residual filling was mapped directly to residual neck or sac presence; micro-Doppler signal preservation for patency of branches and the basilar artery was mapped directly to postoperative stenosis or occlusion; preservation of perforator signal was mapped directly to infarct location in the perforator territory; and neuromonitoring change and recovery were mapped directly to brainstem injury location and functional outcomes following injury.

The microsurgical failure-to-rescue index was defined as the proportion of high-risk events occurring intraoperatively that remained unaddressed or resulted in corresponding postoperative injury. These events included suspected compromise of perforators, loss of perforator signal, impaired patency on indocyanine-green angiography, angiographic compromise of branches, clip-related kinking, postoperative stenosis attributable to clip placement, and intraoperative rupture.

Conformal prediction was implemented as an uncertainty layer using prediction sets generated at specified coverage levels instead of forcing single-label classification decisions. Abstention rules triggered by high-entropy predictions, wide posterior intervals, calibration uncertainty, or excessive distance in similarity space between the index case and nearest analogues were employed as the basis for abstention decisions. Abstention decisions were considered expert-review triggers only.

Risk-adjusted observed-minus-expected cumulative sum, variable life-adjusted display, and funnel-plot templates were specified for future quality monitoring of surgical care based upon expected risk estimated from BTOCC, PAH-S-pre, BPCI, CCR, rupture status, brainstem compression, morphology, and treatment era.

All analyses were conducted in a secure local computational environment using Python (v3.14.7) and R (v4.6.1). The Python environment included NumPy (v2.5.1), pandas (v3.0.5), scikit-learn (v1.9.0), SHAP (v0.52.0), XGBoost (v3.4.0), InterpretML (interpret, v0.7.8), and Matplotlib (v3.11.1). The R environment included rms (v8.1-1), brglm2 (v1.1.0), logistf (v1.26.1), rstanarm (v2.32.2), brms (v2.23.0), glmnet (v5.0), ordinal (v2026.7-26), pROC (v1.19.0.1), dcurves (v0.5.1), mice (v3.19.0), rpart (v4.1.27), randomForest (v4.7-1.2), qcc (v2.7), and irr (v0.85). These packages supported data preprocessing, missing-data handling, penalised and Bayesian regression, machine-learning model development, internal validation, calibration assessment, decision-curve analysis, explainability evaluation, data visualisation, bootstrap analysis, and inter-rater reliability estimation. Computations were performed on a 64-bit local workstation equipped with a 13th-generation Intel Core i7-13620H processor (2.40 GHz; Intel Corporation, Santa Clara, CA, USA) and 16 GB of random-access memory. All analyses were performed locally in accordance with institutional data-protection and privacy requirements.

### 2.5. Model Evaluation

The evaluation of the models employed multiple dimensions that focused on discrimination, calibration, classification behaviour, clinical usefulness, interpretability, reliability, uncertainty, and stability. This approach was chosen since modelling rare-disease surgery requires calibrated, interpretable, and uncertainty-aware predictions rather than accuracy alone.

Discrimination was assessed for binary outcomes using three methods: area under the receiver operating characteristic curve (ROC AUC), concordance index with leave-one-out cross-validation, and precision-recall area under the curve for unbalanced endpoints. The classification behaviour for both binary and multi-class outcomes was summarised using sensitivity, specificity, positive predictive value (PPV), negative predictive value (NPV), balanced accuracy, F1-score and Matthews correlation coefficient. When possible, exact confidence intervals were calculated. Calibration was evaluated using several measures, including calibration intercept, calibration slope, Brier score, integrated calibration index (ICI) and calibration plots with bootstrap confidence bands. In cases where a model had acceptable discrimination but poor calibration, it was classified as statistically informative but clinically unreliable.

Decision-curve analysis (DCA) was used to assess the clinical utility of the model. The net benefit of the model was calculated at a variety of clinically relevant threshold probabilities and compared against “maximum safeguards for all” and “no escalation” strategies. Since these thresholds represented decisions about how to proceed in practice based on the predicted probability of an event occurring, they are referred to here as clinical anchor points. It is important to note that these thresholds represent clinical anchor points and not definitive treatment thresholds.

Ordinal outcomes, including modified Rankin Scale and Glasgow Outcome Scale, were analysed using ordinal c-index, Somers’ Dxy, mean absolute ordinal error, quadratic weighted kappa, and cumulative-probability calibration. The proportionality of odds assumption was evaluated in each case. Partial proportional odds models were developed when this assumption was violated. Favourable dichotomised outcomes, such as modified Rankin Scale 0–2, were also analysed secondarily to provide some level of comparison with other studies within the field of neurosurgery. However, ordinal modelling remained the primary method for evaluating outcomes.

Continuous outcomes, including operative time, temporary occlusion time, blood loss, ICU and hospital length of stay, pontine infarct volume, Montreal Cognitive Assessment, quality-of-life score, and angiographic follow-up duration, were modelled using robust generalised linear models, Bayesian generalised linear models, or quantile regression depending on the underlying distributional characteristics of each outcome.

#### 2.5.1. Global Explainability Methods

Methods to evaluate global explainability of the model included permutation importance, model reliance, accumulated local effects (ALEs), partial dependence plots (PDPs), and bootstrap feature-rank stability. SHAP was used as a local explanation tool when available. Due to high levels of attribution instability in smaller datasets, an Explanation Stability Index (ESI) was developed. The ESI represents the average Jaccard similarity of the most influential features across bootstrap-sampled models. Only explanations that were stable and could be expressed in terms of surgical concepts, such as perforator hazard, brainstem compression, corridor constraint, clip reconstruction burden, rupture severity, temporary occlusion, verification failure, or incomplete rescue, were considered interpretable.

#### 2.5.2. Counterfactual Explainability

Potentially modifiable factors associated with model predictions were explored through counterfactual-style explanations. Examples include reducing temporary occlusion time, improving visualisation of the perforators during surgery, utilising intraoperative digital subtraction angiography, or correcting compromised clips. Each counterfactual was only accepted if supported by temporal order and clinical plausibility.

#### 2.5.3. Uncertainty

Uncertainty was treated as a safety-relevant feature. Bayesian models reported credible intervals and posterior probabilities. Bootstrap models reported optimism-adjusted estimates. Conformal models provided prediction set size and abstention frequency.

#### 2.5.4. Reliability

A major concern when relying upon an individual researcher’s assessment is the potential for variability in assessment. Therefore, it was essential to assess inter-rater reliability using ratings obtained from two neurosurgeons. To accomplish this task, the same 10 patients were rated independently by both neurosurgeons. Both neurosurgeons rated these patients based on preoperative characteristics, including PAH-S-pre and BTOCC components derived from preoperative imaging. The neurosurgeons were blinded to all intraoperative findings, postoperative diffusion-weighted imaging, postoperative angiographic occlusion status, discharge status, mortality, and long-term follow-up. The preoperative ratings were finalised and locked before linkage to postoperative outcomes. Intraoperative assessments and video-based variables were also completed by neurosurgeons who had access to the relevant intraoperative materials; however, they remained blinded to postoperative imaging and clinical outcomes. Postoperative adjudication constructs, including RASP-final, were rated separately using the postoperative information required by each construct. Additionally, the raters’ assessments were kept separate so that neither rater knew the other rater’s initial ratings.

Cohen’s kappa was used to evaluate agreement for nominal variables. Weighted kappa with quadratic weights was used to evaluate agreement for ordinal variables. Composite scores were analysed using an intraclass correlation coefficient model with two-way random effects, absolute agreement, and a single measure [ICC(2,1)]. Bland–Altman analysis was used to assess agreement and systematic differences between continuous measurements made by the two raters. Internal consistency of the rating scales was assessed only when the constituent items were sufficiently homogeneous to represent a common construct.

The reliability estimates were interpreted as reflecting reproducibility under independent and temporally appropriate assessment conditions. Specifically, all preoperative and intraoperative constructs were assessed under outcome-masked conditions, whereas postoperative adjudication constructs incorporated the postoperative information required by their definitions. Reliability estimates were not considered substitutes for external validation or evidence that the measures would retain equivalent reliability when evaluated by different raters, at different institutions, or using different imaging protocols.

All statistical evaluations were performed using two-sided tests with an α level of 0.05. Effect sizes, confidence and credible intervals, calibration, and net benefit were prioritised over *p*-values. Given the limited statistical power and sparse data structure, the findings were interpreted as internally validated exploratory signals requiring external validation before any inference regarding generalisability or clinical deployment.

## 3. Results

### 3.1. Cohort Characteristics and Clinical Outcomes

During the period in which this research was conducted, the researchers evaluated 48 patients with basilar trunk aneurysms who presented to the hospital. Of these, 36 patients underwent open microsurgical treatment for their aneurysms. The researchers identified 31 consecutive patients who met all inclusion criteria as the final group to be analysed. All 31 of these patients underwent either open microsurgical clipping or open reconstructive microsurgery. There were 18 women (58.1%) and 13 men (41.9%) in the patient population, with a median age of 55 years (IQR, 44–63). Fourteen patients (45.2%) had ruptured aneurysms. Among patients whose aneurysms had ruptured, the median Hunt–Hess grade was 2 (IQR, 2–3), and the median Glasgow Coma Scale score upon admission was 14 (IQR, 13–15). Twelve aneurysms (38.7%) were located in the lower basilar trunk, 11 (35.5%) in the middle basilar trunk, and eight (25.8%) in the upper basilar trunk.

Saccular morphology was the most common form of aneurysm, occurring in 17 patients (54.8%), followed by five patients (16.1%) with fusiform morphology, four patients (12.9%) with partially thrombosed morphology, three patients (9.7%) with dissecting morphology, and two patients (6.5%) with giant aneurysm morphology. Median maximum aneurysm diameter was 12.1 mm (IQR, 8.5–16.3), and median neck width was 5.8 mm (IQR, 4.0–9.8).

**Figure 1:**
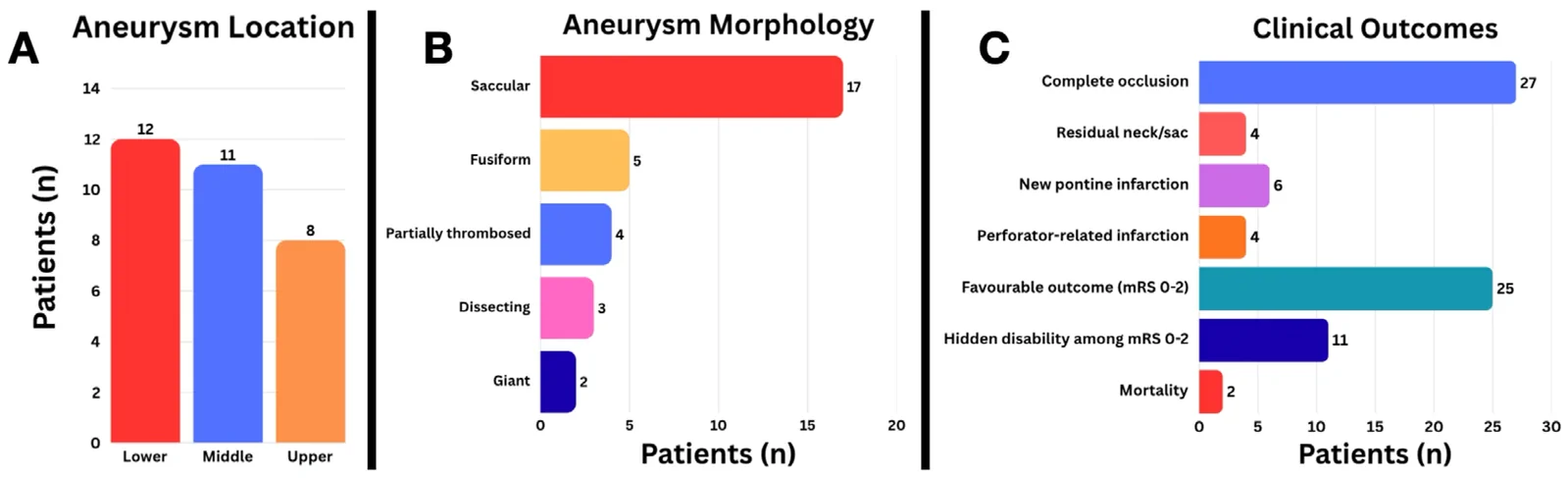
Cohort phenotype and clinical outcome overview. (**A**) Distribution of aneurysm location along the basilar trunk. Lower, middle, and upper basilar trunk aneurysms accounted for 12 (38.7%), 11 (35.5%), and 8 (25.8%) cases, respectively. (**B**) Distribution of aneurysm morphology. Saccular aneurysms predominated, followed by fusiform, partially thrombosed, dissecting, and giant aneurysms. (**C**) Main angiographic and clinical outcomes. Complete occlusion was achieved in 27 patients (87.1%), while a residual neck or sac was present in 4 patients (12.9%). New postoperative pontine infarction occurred in 6 patients (19.4%), including 4 perforator-related infarctions (12.9%). A favourable outcome at the last follow-up, defined as mRS 0–2, was achieved in 25 patients (80.6%); hidden disability was present in 11 of these 25 patients (44.0%). Mortality was 6.5%. Percentages were calculated from the 31-patient cohort unless otherwise specified.

Twenty-seven patients (87.1%) had complete occlusion, three patients (9.7%) had residual neck, and one patient (3.2%) had residual sac. Figure 1 illustrates the frequency of aneurysm location, aneurysm morphology, and critical angiographic and clinical results.

Beginning with new postoperative pontine infarction, which was the primary clinical endpoint, six patients (19.4%) developed a new postoperative pontine infarction. Perforator-related infarctions occurred in four patients (12.9%). Favourable functional status at the last follow-up, as defined by a modified Rankin Scale score of 0–2, was achieved by 25 patients (80.6%). In-hospital, 30-day, and 90-day mortality were each 2/31 (6.5%), and both deaths were due to high-grade rupture. The median duration of clinical follow-up was 24 months (IQR, 12–60; range, 6–120), and clinical follow-up was available for all 31 patients. The median duration of angiographic follow-up was 18 months (IQR, 12–36; range, 6–72), and angiographic follow-up was available for 27/31 patients (three patients had CTA only, and one patient declined additional imaging after complete occlusion had been documented at 12 months). All longitudinal and durability assessments were performed in patients who provided sufficient follow-up data.

Hidden disability was recognised in 11 of 25 patients (44.0%) with favourable modified Rankin Scale outcomes. Hidden disability was most commonly manifested by fatigue in eight of 25 patients (32.0%), executive dysfunction in seven of 25 patients (28.0%), and impaired gait or balance in six of 25 patients (24.0%). These data indicate that clinically relevant morbidity can exist even when technical success of reconstruction is realised and when conventional functional outcome is measured using the modified Rankin Scale.

### 3.2. Derived Microsurgical Intelligence Scores: Distributions and Univariate Signals

Each of the scores developed in this study could be computed from data available for analysis in the final analytical cohort. The range for the SAPIENS-Dataset contribution score was 58 to 86. The median value for the SAPIENS-Dataset contribution score was 74 (IQR, 68–79); 18 of the 31 cases (58.1%) qualified as ready for artificial intelligence development, while seven cases (22.6%) were classified as prototypes for explainable AI. These two classifications formed the predefined strata for AI-development readiness.

The median BTA-SAPIENS-Case score was 78 (IQR, 71–84). BTA-SAPIENS-Case was treated exclusively as a descriptive measure of case-level documentation and learning density. Although its distribution differed between patients with and without postoperative pontine infarction, this association was considered potentially confounded by treatment era, imaging availability, and documentation intensity and was not interpreted as evidence of a protective effect or independent predictive value.

Since potential adverse outcomes in treating basilar trunk aneurysms are likely to result from several pathological processes that might interact with one another, e.g., damage to perforators, compression of brainstem structures by mass effect, limited access for repair, complexity of reconstruction, and failure to perform technically adequate intraoperative rescue manoeuvres, assigning separate dimensionality to the scores enabled classification into separate dimensions that corresponded to clinically meaningful aspects of managing basilar trunk aneurysms. As shown in Table 3, these include hazards related to perforators, vulnerabilities of the brainstem, constraints resulting from the operative corridor, burden of clipping/repair effort, and technical performance after adjustment for rescue manoeuvres.

Unless stated otherwise, all thresholds used in the study were to be considered only as an “exploratory anchor point” and not as a clinically valid decision-making threshold. Therefore, continuous score results were given priority over results that involved dichotomizing data and thus generating hypotheses. The odds ratio, confidence interval, internally derived area under the receiver operating characteristic curve (AUC), and the Spearman’s rho rank correlation coefficient were to be viewed with caution due to both the small number of patients and limited number of endpoints.

There was a statistically significant association for the group of patients who had a PAH-S-pre value greater than 50 vs. those with a PAH-S-pre ≤ 50 and the development of new pontine infarction after surgery (odds ratio = 5.7; 95% CI = 1.3–25.1). Using leave-one-out cross-validation, the LOOCV AUC for the use of PAH-S-pre as a model for predicting new postoperative pontine infarction was 0.85 (95% CI = 0.71–0.96) for each patient. Due to both the exploratory nature of the threshold and the fact that there were only six instances of pontine infarction, this result was considered to be an internal hypothesis-generating signal rather than a valid risk threshold or final estimate of predictive performance.

There was a significant difference between patients who had suffered postoperative pontine infarction and those who had not, such that patients with postoperative pontine infarction had a lower median RASP-final than patients without postoperative pontine infarction (median RASP-final, 72 vs. 84; *p* = 0.003). Because RASP-final includes penalties based upon postoperative harm by definition, the relationship between RASP-final and postoperative pontine infarction status should be interpreted as a technical-performance endpoint signal rather than evidence of independent predictive capacity concerning the outcome prior to surgery.

Furthermore, the association between BPCI and both preoperative brainstem symptoms and previously undetected hidden disability suggests that proximity and compression of brainstem structures remain considerations that must be taken into account regardless of whether additional intervention will be required in evaluating successful angiographic reconstruction.

Finally, CCR correlated with operative time, clip repositioning, and surgeon-rated complexity, which supports its utility as an index of restriction in terms of accessibility. Lastly, CRB-predictor was associated with lower odds of achieving complete occlusion and longer operative time, consistent with its role as a measure of burden associated with reconstructive efforts.

### 3.3. Primary Clinical Endpoint: New Postoperative Pontine Infarction

A new postoperative pontine infarction occurred in six patients. In the exploratory Firth penalised logistic regression model, a relationship was identified between PAH-S-pre and new pontine infarction (odds ratio [OR], 1.19 for every 5-point increase; 95% confidence interval [CI] corrected using the Firth method, 1.04–1.36), as well as an additional relationship between BPCI and new pontine infarction in the parsimonious model containing only the two predictors (OR, 1.38 for every 0.2-unit increase; 95% CI, 1.02–1.87). These estimates were unstable given that there were only six events and two predictor parameters; therefore, they should be viewed as preliminary, hypothesis-generating signals rather than as independently validated effects.

Each LOOCV iteration was initiated before conducting any data-dependent preprocessing steps or estimating the models. All imputation, scaling, feature selection, threshold derivation, hyperparameter selection, calibration, and model fitting were completed using only the 30-patient training set. The patient removed from the dataset during each cross-validation iteration was completely excluded from all data-dependent steps until the final prediction was made. Score definitions and weights were determined separately before the outcome analyses and were therefore not re-estimated during the cross-validation iterations.

The internal estimate of the LOOCV AUC was 0.92 (95% CI, 0.80–0.99), and the Brier score was 0.08. Estimates of the calibration intercept and slope were provided as descriptive results because it was not possible to perform meaningful calibration assessments with only six endpoint events. Decision-curve findings were similarly considered exploratory and were therefore not used to identify clinical thresholds or guide treatment strategies.

**Figure 2:**
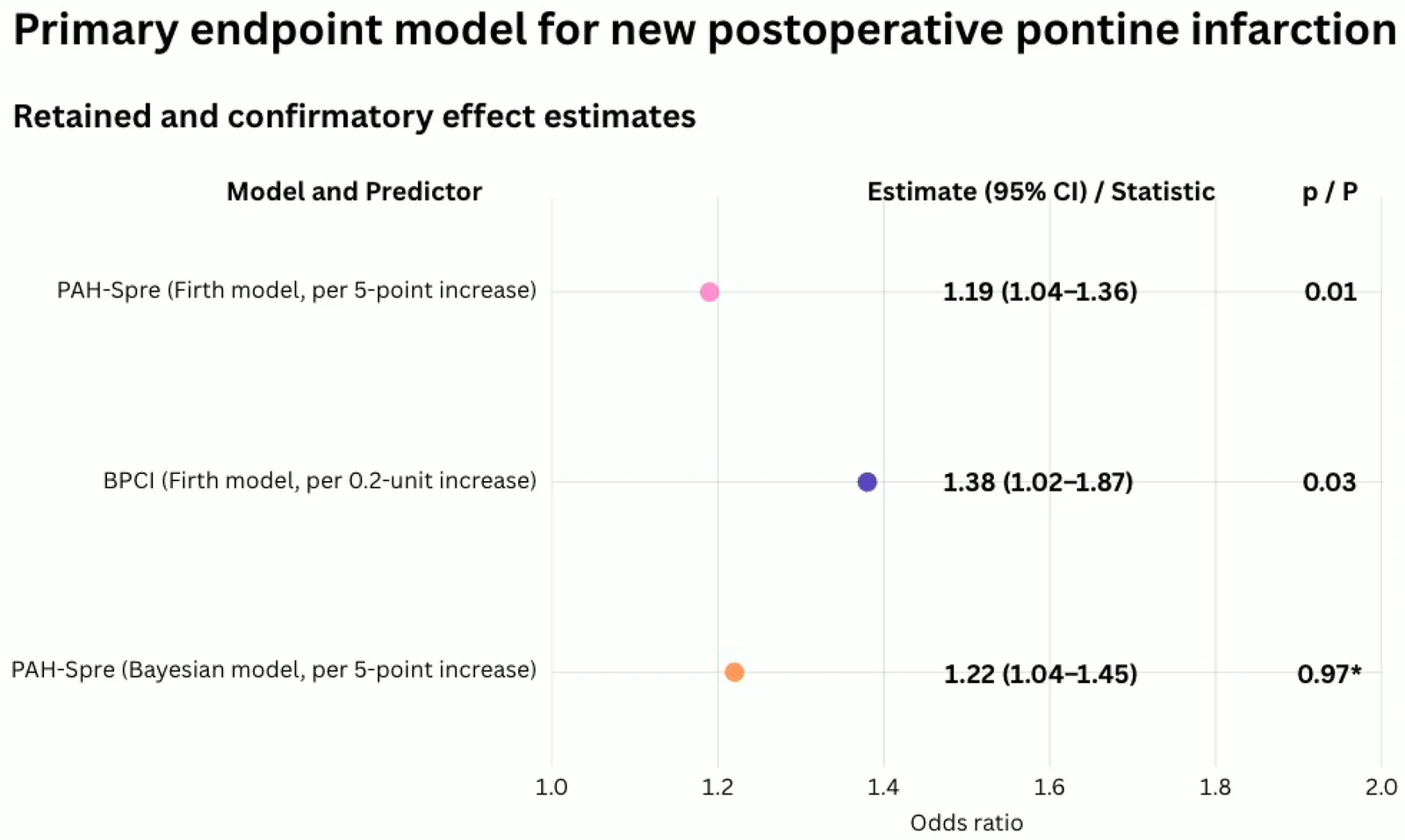
Primary endpoint model for new postoperative pontine infarction. Forest plot showing retained and confirmatory effect estimates for the primary endpoint model. PAH-S-pre retained an independent predictive signal in the prespecified Firth penalised logistic regression model, with an OR of 1.19 per 5-point increase (95% CI, 1.04–1.36; *p* = 0.01). BPCI also retained predictive value, with an OR of 1.38 per 0.2-unit increase (95% CI, 1.02–1.87; *p* = 0.03). Bayesian logistic regression with weakly informative priors supported the PAH-S-pre signal, with a posterior median OR of 1.22 per 5-point increase, a 95% credible interval of 1.04–1.45, and a posterior probability of 0.97 that the OR exceeded 1.10. * Posterior probability that the OR exceeded 1.10.

Bayesian logistic regression was used as a sensitivity analysis rather than as separate, independent confirmation. The posterior median OR for PAH-S-pre was 1.22 for every 5-point increase in PAH-S-pre (95% credible interval, 1.04–1.45), with a posterior probability of 0.97 that the OR exceeded 1.10. Of the six pontine infarctions, four were adjudicated as being perforator-related. The consistent direction of the effects observed in both the Firth and Bayesian analyses supports continued investigation of the perforator-aware construct; however, it does not establish either external validity or clinical utility. Both estimates derived from the Firth regression analysis and the Bayesian sensitivity estimate are presented in Figure 2. Additionally, the consistent direction of the Firth and Bayesian estimates was compatible with the biological plausibility of the perforator-aware construct and its potential relevance to basilar trunk aneurysm surgery, where injury to small perforating vessels can result in considerable morbidity.

### 3.4. Secondary Endpoints: Occlusion, Functional Outcome, and Hidden Disability

Exploratory Firth regression modelling identified associations with predictors of complete occlusion, including CRB-predictor, neck width, and fusiform morphology. Each additional 5 points of CRB-predictor was associated with decreased odds of complete occlusion (OR per 5 points, 0.85; 95% CI, 0.73–0.97; *p* = 0.02), as was increased neck width (OR per mm, 0.78; 95% CI, 0.61–0.97; *p* = 0.03). The greatest morphological penalty for complete occlusion was associated with fusiform morphology (OR, 0.21; 95% CI, 0.04–0.94; *p* = 0.04). The complete occlusion model exhibited acceptable leave-one-out cross-validated AUC of 0.86 (95% CI, 0.74–0.96).

Residual neck or sac filling was noted in four patients (12.9%) and was associated with elevated levels of CRB-predictor values (OR per 5 points, 1.18; 95% CI, 1.03–1.36), as well as fusiform or dissecting morphology (OR, 4.2; 95% CI, 1.1–16.0). While these findings suggested that residual filling was more directly related to reconstructive challenge and non-saccular morphology rather than aneurysm size alone, the number of residual filling lesions was too few to allow meaningful inference regarding this relationship. Clinically, CRB-predictor values appear to reflect a unique technical burden that may be important to incorporate into operative planning, intraoperative verification, and early angiographic follow-up.

Ordinal Bayesian regression modelling allowed us to retain the full structure of the modified Rankin Scale to assess functional outcomes. Elevated values of PAH-S were associated with poorer modified Rankin Scale category (OR per 5 points, 1.12; 95% credible interval, 1.02–1.24), as was new pontine infarction (OR, 5.1; 95% credible interval, 1.3–20.2). Values of RASP-final that were higher than those in the lower range of the distribution produced shifts in the posterior distributions toward better functional outcome, whereas lower values of RASP-final produced posterior distributions that were skewed toward greater disability risk (inverse OR for improvement, 0.91; 95% credible interval, 0.84–0.98). The ordinal c-index was 0.84.

Of the 25 patients who ultimately had favourable outcomes with a final modified Rankin Scale ranging from zero to two, hidden disability was associated with BPCI > 0.5 (OR, 7.2; 95% CI, 1.5–35.8), pontine infarction (OR, 6.3; 95% CI, 1.1–36.2), and cranial nerve contact burden (OR per grade, 1.5; 95% CI, 1.0–2.2). Therefore, anatomical brainstem vulnerabilities and minor ischaemic injuries may contribute to recovery beyond what is apparent through traditional measures of global disability, even after a patient achieves favourable outcomes with respect to these measures.

### 3.5. AI Model Performance, Validation, and Explainability

Failure of the technical aspects of surgery occurred in eight out of 31 cases (25.8%) and included a combination of factors such as insufficient closure; compromised blood supply through the parent artery, branch, or perforator; new postoperative pontine infarction; or poor functional outcome at the time of discharge, as defined by an mRS score greater than two. In order to predict the likelihood that technical failure would occur when using the Firth model, predictor variables included PAH-S-pre, BPCI, CCR, CRB-predictor, and rupture status. The number of events (eight) divided by the number of predictor parameters (five) resulted in 1.6 events per predictor parameter. This sparse information density, despite employing Firth penalisation together with fully nested leave-one-out cross-validation, resulted in potentially unreliable multivariable predictions. Therefore, each performance metric was presented as an exploratory and potentially unstable internal estimate rather than as a validated measure of individual-level predictive performance.

Each LOOCV iteration was initiated prior to any data-dependent preprocessing or model estimation. The internally estimated AUC for the Firth model was 0.91 (95% CI, 0.83–0.97); balanced accuracy was 0.84; the F1 score was 0.79; and the MCC was 0.68. The calibration intercept was −0.08; the calibration slope was 0.96; the Brier score was 0.10; and the integrated calibration index was 0.045. Because only eight events occurred, calibration metrics were reported on a descriptive basis only. After adjusting for optimism via bootstrapping, the AUC was estimated at 0.88 (95% CI, 0.79–0.95). The permutation test indicated *p* < 0.01; however, the limitations inherent in the small cohort size, low event count, and derivation of all constructs within the same cohort limited interpretation.

Internally estimated AUC values for the random forest and monotonic XGBoost models ranged from 0.89 to 0.90. Calibration slopes for the two models were 0.85 and 0.86, respectively, while the Brier score for both models was 0.13. Limited information density precluded interpreting differences between the Firth model and the other models as evidence of superior algorithmic performance.

The Explainable Boosting Machine produced visually informative representations of the associations between estimated risk, PAH-S-pre, and CCR. Estimated risk increased with increasing PAH-S-pre values above approximately 40 and increasing CCR values above approximately 1.5. These trends were viewed as exploratory representations of how well the model fitted the data rather than as validated biological or clinical thresholds. Similarly, PAH-S-pre and CCR cut-off values utilised throughout these analyses were regarded as exploratory reference points and were not used to produce continuous-score AUC estimates.

A shallow classification tree fitted to the entire cohort established an exploratory phenotypic association that combined PAH-S-pre > 53 and CCR > 1.6. Technical-safety failure occurred in five of six patients satisfying this rule, whereas three of 25 patients failing to satisfy this rule experienced technical-safety failure. As no independent cross-validation was performed for tree construction, split selection, or threshold determination, these values reflected apparent full-cohort performance only. Thus, this rule was considered merely descriptive and hypothesis-generating, and additional studies will be required to validate the accuracy of this rule and establish the validity of the treatment thresholds and its clinical utility.

Summary statistics for the internal estimations and nonlinear benchmarking studies, along with the extraction of rules from the exploratory analysis, are summarised in Table 4. These studies were intended to illustrate the proposed framework and should not be taken as evidence of external validation or suitability for clinical use.

Conformal prediction at a target coverage level of 90% showed that average prediction set size was equal to 1.2; abstention due to uncertainty occurred in four patients (12.9%), and among them, 2 patients (50%) underwent revision of preliminary algorithmic risk categorisation through expert adjudication. There is no general consensus on whether and how to use conformal prediction to direct patient management decisions or clinical practice recommendations; however, our data suggest that estimating uncertainty may act as a trigger prompting senior review rather than serving as an autonomous decision-making mechanism.

Global SHAP analysis determined that the top explanatory variables driving model predictions were PAH-S-pre, BPCI, and CCR, with mean absolute SHAP values representing each variable being equal to 0.28, 0.19, and 0.15, respectively. Additionally, the Explanation Stability Index for the top three features over all bootstraps (n = 2000) was equal to 0.81 (95% confidence interval, 0.76–0.86). Local explanations translated into surgically meaningful terms; specifically, high-risk predictions were associated with combinations of high-density perforators adjacent to the neck, large brainstem proximity-compression burden, geometric constraints on corridor burden, and high degrees of corridor constraint.

The surgical digital twin model achieved a top-one nearest-neighbour concordance rate of 74.2% and top-three majority accuracy rate of 83.9% for the primary outcome measure. Concordance rates for approach choice, clip strategy, and DWI outcome were reported to be 87%, 71%, and 77%, respectively. The three cases exhibiting distances larger than the 90th-percentile threshold also triggered uncertainty abstention in all instances, indicating that the similarity model identified anatomy falling outside typical patterns of operative action described within the registry.

### 3.6. Exploratory Comparison with the De Novo BTOCC Construct and Reliability

BTOCC was created from the data in this study and was therefore used for a comparison to the internally developed additional metrics (corridor constraint, reconstruction burden, etc.), and as such should be viewed as exploratory and not as comparative to an existing classification system. As only four perforator-related ischemic events occurred, we did not feel that it would be statistically valid to make claims about the incremental predictive value of these metrics; nor could we accurately estimate their ability to improve risk prediction through metrics like NRI, IDI, or CINB.

These supplementary metrics are useful as descriptive representations of specific hazards associated with the surgical procedure (perforator hazard, brainstem vulnerability, corridor constraint, and reconstruction burden). The associations between the supplementary metrics and BTOCC can provide hypotheses regarding possible additional independent factors contributing to surgical complexity rather than provide evidence that they are better than an established classification system.

Interobserver agreement between neurosurgery experts evaluating 10 randomly selected patients demonstrated substantial agreement regarding both perforator incorporation grade (weighted kappa statistic = 0.78) and video-based perforator grade (kappa statistic = 0.72); total PAH-S (intraclass correlation coefficient = 0.88); and total BTOCC (intraclass correlation coefficient = 0.84), providing support for the reproducibility of the semi-quantitative anatomical and operative rating systems used in the population studied herein.

### 3.7. Longitudinal Outcomes, Durability, and Sensitivity Analyses

Median modified Rankin Scale scores improved significantly from hospital discharge median mRS = 2 to last follow-up median mRS = 1 (Wilcoxon signed-rank test, *p* = 0.01). Median modified Rankin Scale scores improved by ≥1 grade in 12 patients (38.7%), remained unchanged in 16 patients (51.6%), and worsened in three patients (9.7%).

Ordinal regression adjusted for age at treatment, rupture status, and PAH-S values showed a common odds ratio for better outcome at last follow-up equal to 2.3 (95% Bayesian credible interval, 1.2–4.1).

Estimated retreatment-free survival was equal to 93.5% at 12 months post-treatment and 90.3% at 24 months post-treatment.

No clip migration or delayed parent-artery stenosis was observed among the 27 patients with available follow-up vascular imaging. Approximately 92.6% of patients treated initially with complete occlusion maintained stable complete occlusion at last angiographic follow-up evaluation, indicating favourable angiographic durability of open reconstruction techniques utilised in treatment of the patient population studied herein.

Temporal trend analysis revealed a decreasing incidence of new pontine infarction from 30% before 2010 to 17% during 2010–2019 and 0% during 2020–2025 (*p* = 0.04). Conversely, RASP-final increased from 72 before 2010 to 82 during 2010–2019 and 88 during 2020–2025 (*p* = 0.01). Trends observed were consistent with evolution toward increasingly refined practices concerning case-selection criteria, definitions used to define disease severity at diagnosis, procedures employed for verification during surgery, techniques utilised for preservation of perforators during surgery, rescue preparation, and clip-reconstruction techniques; however, caution must be exercised when analysing temporal trends due to the limited number of cases within each era.

## 4. Discussion

### 4.1. Principal Findings and Interpretive Frame

A fundamental objective of this study was to develop an intelligent, explainable framework for microsurgical management of basilar trunk aneurysms treated by open clipping or open reconstructive microsurgery. Unlike many conventional predictive approaches, the key contribution of this study was to make expert microsurgical reasoning transparent, organised temporally, and analytically testable.

There are numerous variables and interactions that determine outcomes in this area. Variables that contribute to outcomes include anatomic features of the aneurysm; anatomy of the perforators; proximity to the brainstem; geometric limitations of the corridor; need for clip placement vs. reconstruction; intraoperative verification of results; need for rescue manoeuvres; and the patient’s remaining postoperative neurologic reserve [17]. Essential descriptors, including aneurysm size, ruptured/unruptured status, occlusion grade, mortality rate, and modified Rankin Scale score, are critical for determining the rationale for surgical intervention for a basilar trunk aneurysm; however, they do not define operative reasoning [18].

Development of this novel framework helps fill gaps in our understanding. The framework defined four core operative domains for basilar trunk aneurysm surgery: perforator-aware hazards; brainstem proximity/compression burden; operative corridor constraints; and clip/reconstruction burden. These four operative domains were complemented by four additional interpretive and readiness constructs: rescue-adjusted surgical precision; learning density per case; readiness of the dataset; and reference operative complexity.

Separation of the domains was required to establish that adverse outcomes associated with treatment of basilar trunk aneurysms can result from different mechanisms despite sharing common labels. As an example, a new pontine infarction can occur secondary to several mechanisms, including perforator inclusion; clip-line crossing; temporary occlusion; kinking due to perforator location; poor visualisation; or late thrombosis. Another example is a residual sac that can occur due to a combination of factors, including limited reconstructive capability; fusiform shape of the aneurysm; priority for preserving the parent vessel; or deliberate safety compromise [19].

While patients achieving a favourable modified Rankin Scale outcome can have good global functional outcomes, they may experience long-term fatigue, disequilibrium, a decrease in cognitive function, and decreased ability to return to work. Since global metrics cannot differentiate technical success from persistent subtle morbidity, the proposed score design was developed to maintain utility as a surgical measure [20].

Each of the derived constructs demonstrated coherent and clinically relevant signals within this population. PAH-S-pre showed an exploratory internal association with new postoperative pontine infarction and thus supported the biological relevance of prior knowledge of perforator anatomy. BPCI demonstrated a correlation with brainstem syndrome and hidden disability, suggesting that proximity and compression of the brainstem may affect recovery beyond what is measured by traditional functional assessments. CCR demonstrated a correlation with operative time, clip repositioning, and surgeon-rated complexity, suggesting that it is a useful measure of access constraint. CRB-predictor demonstrated an association with complete occlusion and operative burden, supporting its definition as a metric of reconstructive demand. RASP-final demonstrated associations with both technical performance and harm burden while being appropriately categorised as an endpoint and not as an independent predictor.

The composite technical-safety failure model produced exploratory internal estimates of discrimination, calibration, and interpretability; however, these estimates remained potentially unstable because only eight events were available for five predictor parameters.

A total of 87.1% of patients achieved complete occlusion, 80.6% had favourable functional outcomes at final follow-up, and 6.5% died in our cohort. A systematic review and meta-analysis by Tian et al. comparing open and endovascular treatment of basilar trunk aneurysms found that open surgical procedures yielded a pooled favourable-outcome proportion of 46%, whereas endovascular therapy yielded a pooled favourable-outcome proportion of 75%, with considerable heterogeneity [3]. However, direct comparisons between open and endovascular treatment demonstrated no statistically significant differences in favourable outcome, mortality, complications, or complete occlusion. Gu et al. reported a favourable discharge outcome in 53.6% of their intervention group, which included primarily endovascularly treated patients, with one patient undergoing bypass, and a mean follow-up of 23.23 ± 21.58 months. Therefore, although the results from our cohort appear favourable when considered alongside other cohorts in the literature, we do not consider direct comparison appropriate because of marked differences in aneurysm morphology, rupture status and severity, treatment selection, institutional expertise, timing of outcome assessment, endpoint definitions, and length of follow-up [5]. Our series includes a highly selected population of patients treated by open microsurgery and therefore cannot be interpreted as implying superiority over either endovascular therapy or conservative management.

The findings should be viewed as internally validated proof-of-concept signals rather than as evidence supporting deployment of a clinical product. The primary contribution of these findings is that they illustrate that rare and severe forms of cerebrovascular surgery can be modelled using structured variables proximal to surgical anatomy and decision-making processes. Rather than suggesting that AI supplants expert decision-making, this study illustrates that expert decision-making can be converted into a replicable structure and possibly facilitate learning, risk formulation, quality assessment, and future externally validated decision-support research.

### 4.2. From Descriptive Aneurysm Reporting to Mechanism-Specific Microsurgical Modelling

Basilar trunk aneurysms represent one of the most surgically challenging subsets of cerebrovascular surgery. In order to minimise damage to small perforators supplying compact brainstem regions, deep exposures must be carefully planned. Additionally, reconstructed parent vessels must preserve cranial nerves and verify blood flow in regions where even minor errors can result in significant morbidity.

Historically, the majority of the literature detailing aneurysm surgery has focused on general descriptors such as rupture status, anatomical size, morphology, occlusion grade, morbidity, mortality, and global functional outcome. While use of these general descriptors remains essential for comparing studies, they fail to describe the anatomical and technical considerations necessary for determining microsurgical success [21].

As part of this study, an analytical language was developed for transitioning from reporting descriptive outcomes to modelling mechanism-specific components.

In essence, a basilar trunk aneurysm can be characterised as “difficult” due to multiple reasons. Reasons include: perforators may originate from either the neck or dome of the aneurysm; clips may cross over a branch-bearing surface; domes may compress the pons; corridors may be narrower than the depth of the lesion; lesions may require fenestrations or tandem reconstructions; or intraoperative verification may necessitate compromises reducing optimal procedural completion.

Each reason presents unique challenges in terms of planning, exposure, clipping, verification, rescue, and follow-up. The framework separates these into four core operative domains and additional interpretive constructs: perforator-aware hazards (PAH-S); brainstem proximity/compression burden (BPCI); corridor constraint ratio (CCR); clip/reconstruction burden (CRB); rescue-adjusted surgical precision (RASP); learning density per case (SAPIENS); dataset readiness (SAPIENS-Dataset); and reference operative complexity (BTOCC). Separation into these domains was essential since complexity affects surgical pathway decisions before surgery begins, during surgery, and after surgery.

For instance, a perforator-dominant case may present different needs for preoperative preparation compared to a corridor-dominant case. Also, an aneurysm causing compression to the brainstem may require different counselling and follow-up care compared to an aneurysm that is morphologically complex but causes no compression to the brainstem. Further, a case requiring extensive reconstructive effort may involve elaborate planning for clip placement(s), intraoperative angiography, and early postoperative angiographic surveillance. Lastly, cases requiring high levels of intraoperative rescue manoeuvring may be technically successful but offer abundant opportunities for education related to reviewing complications [22].

Therefore, whereas previous studies asked whether a case was difficult, this framework asks why it was difficult and at what point during the operative process the difficulty occurred [23].

Additionally, separation in terms of timing was necessary. Contamination can occur in predictive models utilising surgical data if postsurgical injury, final angiographic results, or rescue outcomes impact preoperative predictions. This phenomenon is especially relevant in small datasets where few outcome-linked variables can generate seemingly strong yet clinically meaningless results. PAH-S-pre and PAH-S-post were separated from PAH-S-intra so that the same clinical domain could be represented throughout all phases of the operational period while preserving analytic distinctions between prediction, intraoperative updates, postoperative evaluation, and assessments of technical proficiency.

### 4.3. Contemporary Treatment Context and Morphological Complexity of Basilar Trunk Aneurysms

BTAs represent an important group of aneurysms treated using endovascular techniques. However, there are still significant concerns regarding both procedural complications and the potential lack of long-term durability. In a recently published study in which 90 patients were followed for a median of 51 months after treatment, favourable outcomes were achieved in 75.6% of patients [24]. The reported complications included ischaemic events in 25.6% of cases, mortality in 8.9%, and an overall complication rate of 33.3%. Follow-up imaging revealed that complete occlusion was achieved in 71.7% of patients, whereas recanalisation occurred in 4.3% and aneurysm enlargement in 4.3%. These data suggest that, although achieving a satisfactory initial treatment result is important, it does not obviate the need for ongoing clinical and radiographic evaluation [25].

Due to the large size of many BTAs, they are often particularly challenging to manage using endovascular techniques. Many BTAs can extend across a broad portion of the parent artery, and this may complicate management because of mural thrombus at the site of the lesion, perforating branches arising from the aneurysmal sac, and persistent mass effect on adjacent brainstem structures, which may limit the ability to achieve immediate and complete aneurysm exclusion [7]. Due to the complexity of endovascular treatment, additional techniques may be necessary, including the placement of overlapping stents, the use of flow-diversion devices, adjunctive coiling procedures, and/or parent-artery occlusion. Although these additional technical manoeuvres provide further options for managing BTAs, they also introduce risks such as perforator infarction, thromboembolic events, haemorrhage, in-stent or parent-artery thrombosis, incomplete occlusion, recanalisation, repeat intervention, and prolonged antiplatelet therapy. For example, in a report involving 62 large vertebrobasilar trunk aneurysms, the overall complication rate was 16.1%, complete occlusion was demonstrated in 77.8% of patients undergoing angiographic follow-up, and 5.6% required repeat intervention for recanalization [26].

In addition to presenting clinically with haemorrhage or rupture, BTAs can occasionally present through less common non-haemorrhagic manifestations, including brainstem stroke. Several different mechanisms may contribute to the development of cerebral ischaemia secondary to BTAs. These include compromise of perforating arteries supplying the brainstem, intraluminal thrombus formation within the aneurysm leading to distal embolisation, parent-artery occlusion, and physical distortion or compression of adjacent brainstem structures due to local mass effect or secondary changes produced by previous interventions such as clipping or stenting [27]. For example, Kavak et al. described a 27 mm chronic mural bleeding ectasia associated with extensive thrombosis and severe brainstem compression, producing acute brainstem infarction and demonstrating how BTAs can lead to severe ischaemic events despite not having ruptured [2].

Saccular BTAs form a morphologically distinct subset whose behaviour may vary depending upon their longitudinal location along the basilar trunk, the degree of lateral or posterior dome projection, neck diameter, wall irregularity, the presence of vascular anomalies elsewhere within the arterial tree, and the proximity of perforators to the neck or sac [28]. In one study, 41% of saccular BTAs projected laterally on anteroposterior angiographic views, 32% were associated with multiple aneurysms, and vascular anomalies were frequently identified. Similarly, natural-history studies have shown that saccular BTAs are commonly associated with multiple intracranial aneurysms. These anatomical characteristics may affect operative exposure, clip-path selection, reconstruction requirements, perforator preservation, and suitability for endovascular treatment [8,29].

### 4.4. Perforator-Aware Hazard and Pontine Infarction

The most direct mechanistic signal in this study was the relationship between preoperative knowledge of perforator anatomy and new postoperative pontine infarcts.

This association is biologically plausible and clinically relevant. Perforators arising from basilar segments provide compact brainstem territories in which small ischaemic lesions can create disproportionate neurologic consequences. During basilar trunk aneurysm surgery, perforators can be damaged through several mechanisms: becoming entrapped in the neck of the aneurysm; originating from the sac of the aneurysm; adhering to the dome of the aneurysm; being located posteriorly/laterally, thereby obscuring them from visual inspection; crossing the operative field, thereby competing with clip placement(s); kinking after reconstruction; thrombosing after temporary occlusion; or diminishing flow before closure [30].

PAH-S-pre was designed to evaluate this hazard before damage occurs. There is benefit to incorporating multiple factors that are commonly mentally evaluated by experienced cerebrovascular surgeons during preoperative review and exposure planning. Factors include number of visible perforators; distance to neck; incorporation into the neck; number of perforators originating from the sac; branching cluster(s); potential conflict with clip placement(s); territory at risk; and degree of certainty in assessment. These variables are not abstract concepts but relate directly to decisions made during preoperative review and operative exposure. Therefore, high values of PAH-S-pre can reasonably justify further analysis using three-dimensional angiography, more precise mapping of perforators, more detailed planning of clip placement(s), preparation for alternative verification techniques, and lower threshold for early postoperative diffusion-weighted MRI.

PAH-S-intra extends this concept into the operating room. Preoperative imaging provides clues about possible perforator anatomy; however, additional information is gained once exposed operatively: adherent branch(es); hidden perforator(s) located behind or beneath the neck of the aneurysm; branch(es) crossing over the trajectory of clips applied to control haemorrhage from the aneurysm; transient micro-Doppler changes observed during clipping manoeuvres; abnormal ICG dye distribution indicating insufficient distal perfusion following application of clips to control haemorrhage from the aneurysm; or necessity for repositioning of previously applied clips. All of these findings cannot be analysed using solely preoperative-prediction-based models but are very relevant for updating perioperative risk assessments and formulating plans for rescue procedures.

PAH-S-post was intended for a different purpose than PAH-S-intra. PAH-S-post was designed to aid clinicians with postoperative judgement regarding mechanism for injury to perforators and was never designed to predict those injuries. Distinguishing PAH-S-pre, PAH-S-intra, and PAH-S-post is crucial for establishing credibility in architecture-based scores.

Because postoperative information cannot affect preoperative predictions, PAH-S scores are clinically continuous throughout time. Therefore, PAH-S represents a flexible construct applicable throughout the narrow domain of basilar trunk aneurysm surgery. PAH-S can be used as guidance for preoperative counselling; as guidance for multidisciplinary planning meetings; as support for intraoperative verification strategies; as a surveillance tool via DWI following surgery; and as a template for performing morbidity reviews after basilar trunk aneurysm surgery.

### 4.5. Brainstem Vulnerability and Hidden Disability Beyond Conventional Outcome Scales

Unlike typical methods of measuring aneurysm rupture and complications related to ischaemia, BPCI examines the interaction between the aneurysm and the brainstem. For individuals who undergo surgery on the basilar trunk, morbidity may not only come from rupture and/or procedural ischaemia, but also from other factors, such as contact between the aneurysm and the ventral pons, compression of the pons, oedema, hydrocephalus, deformation of the midbrain, encroachment on the fourth ventricle, or involvement of the cranial nerves. Each of these factors can potentially have an impact on the patient’s presenting symptoms, tolerance to operative manoeuvres, postoperative recovery, and ultimately the patient’s functional capacity postoperatively.

One of the clinically significant correlations observed in this study regarding BPCI was hidden disability. Many cerebrovascular studies define successful outcomes using modified Rankin Scale (mRS) scores of 0–2. The mRS is a reliable and widely adopted method for evaluating the effectiveness of interventions for cerebrovascular disease [31]. However, it does not completely capture the full range of deficits that a patient may have, particularly related to fatigue, poor balance, decreased executive functioning, swallowing difficulties, behavioural problems, decreased physical or mental stamina, or failure to resume previous professional and social roles. Posterior fossa or brainstem-adjacent pathology can cause subtle levels of dysfunction that can significantly impair a patient’s ability to function regardless of whether the patient remains independent [32].

Therefore, identifying hidden disability in patients with favourable mRS scores highlights an important clinical problem: achieving angiographic success and global functional independence does not necessarily translate into full recovery. Even if a patient has achieved both goals angiographically and functionally, it is possible that there could be meaningful limitations that can negatively affect the quality of life of the patient [33].

Therefore, BPCI may provide value not only as a modelling tool but also as a counselling tool and follow-up tool. A higher value of BPCI may encourage treating clinicians to consider further evaluations of a patient’s gait and balance, cognitive function, fatigue level, swallowing issues, mood changes, cranial nerve function, and return-to-work capacity. Thus, BPCI fills the gap between successfully reconstructing a basilar trunk aneurysm and restoring brainstem function and assisting in the reintegration of the patient.

In this sense, BPCI adds to the uniqueness of this research. Most research concerning aneurysms focuses on outcomes primarily related to occlusion rates, mortality rates, and mRS. By focusing on the relationship between aneurysm–brainstem anatomy and the functional burden on the patient, this research expands the concept of success from solely reconstructing vessels to patient-centred recovery.

### 4.6. Corridor Constraint, Reconstruction Burden, and Rescue-Adjusted Performance

CCR and CRB-predictor provide quantifiable measures of two common challenges associated with performing surgery on the basilar trunk that are recognised by neurosurgeons but rarely measured objectively: access difficulty and reconstruction burden.

CCR is an objective quantification of the geometric relationship between aneurysm depth and working corridor width. When CCR is high, surgeons must operate within a narrow corridor at considerable depth with very limited visualisation. Visualisation is limited by several mechanisms, including limited bimanual manoeuvrability, a compromised clip-applier trajectory leading to less-than-optimal inspection of perforators, potentially compromised proximal/distal control, fewer options for rescue manoeuvres, and longer operative times due to clip repositioning. The correlation between CCR and increased operative time, clip repositioning frequency, and surgeon-rated operative complexity provides strong clinical face validity for CCR. CCR converts the subjectively stated “the corridor was difficult” into a measurable parameter. Parameters like CCR can assist neurosurgeons in selecting approaches; determining whether skull-base extension is needed; anticipating areas with limited visualisation; developing clip arrangement strategies; considering endoscopic assistance; and establishing criteria for intraoperative angiography.

CRB-predictor reflects the burden required to repair the segment of the basilar artery containing the aneurysm. Basilar trunk aneurysms usually cannot be repaired by simply closing off the neck. An aneurysm that is wide-necked, fusiform, dissecting, or partially thrombosed; has calcifications; incorporates branches; or is large may require deployment of multiple clips; construction of fenestrated clip constructs; clipping with tandem or stacked configurations; modification or reconstruction of parent arteries; repositioning of clips; wrapping; trapping; or bypass assistance. The correlation between CRB-predictor and complete occlusion, residual filling, and operative time demonstrates that CRB-predictor measures a technical burden that cannot be evaluated by either aneurysm size or rupture status alone.

It is necessary to distinguish CRB-predictor from CRB-final. Since CRB-predictor does not include residual neck/sac filling, CRB-predictor is a pre-outcome measurement of reconstruction burden. On the other hand, CRB-final includes descriptive residual morphology after outcome is determined. This avoids circular logic while maintaining the value of postoperative technical review. Clinically speaking, CRB-predictor may aid in preparing and verifying efforts, whereas CRB-final may assist in understanding final reconstruction and contribute data toward future learning.

RASP introduces dimensions of execution and rescue. Complex aneurysm surgery often occurs with transient events that are not always harmful if identified early and addressed appropriately. Transient events that occur during surgery may include clip-induced narrowing being revised; suspected compromise to a perforator leading to repositioning; abnormalities visible on intraoperative indocyanine green angiography prompting additional examination; changes visible with micro-Doppler prompting adjustments; and monitoring abnormality resolution after correction is made. These events are not entirely represented by final occlusion status alone. RASP-execution captures the intraoperative event prior to postoperative imaging, whereas RASP-final captures final harm/burden from technical failure.

Using RASP in this way ensures that it is used correctly. RASP-final should never be used independently to predict postoperative injuries, as postoperative injury is part of the definition for RASP-final. The usefulness of RASP-final lies in providing a systematic means for evaluating technical performance, verification efforts, rescue manoeuvres, and final burden of harm. Using RASP in this way allows for evaluation of technical performance as part of morbidity conferences, educational programmes for complex aneurysm repair, technical auditing, and quality-improvement initiatives adjusted for risk.

Collectively, CCR, CRB, and RASP convert an analytical paradigm that is concerned with characterising static aspects of aneurysms into a surgical-process modelling paradigm. CCR, CRB-predictor, and RASP facilitate not only characterisation of what the aneurysm was but also what the surgery required, including restrictions encountered during surgery and how intraoperative risks were mitigated.

### 4.7. Explainability, Uncertainty, and Case-Similarity Retrieval

High-risk surgeries like surgery for rare aneurysms require more than just good performance from models: they require explainability, calibration, recognition of uncertainty, and explicit deferral to clinical judgement. Therefore, we emphasised transparency over automation in creating this framework.

The hierarchy of explanation developed by the authors was logically consistent with clinical practice. PAH-S-pre reflected perforator hazard; BPCI reflected brainstem vulnerability and hidden disability risk; and CCR reflected corridor constraint. These parameters are surgically logical as opposed to statistically opaque artefacts. Moreover, explanation stability added another layer of evidence supporting internal consistency of explanations across repeated resampling exercises. Feature attribution can be unstable in smaller datasets; therefore, explanations are not sufficient merely because they seem plausible in one model fit. The authors considered explanation stability as part of model evaluation as opposed to a superficial addition.

Conformal prediction provided a practical dimension of uncertainty. Instead of providing a singular confident classification for each patient, as a single-label classifier would provide, conformal predictors allow for abstention when uncertainty exceeds threshold limits [34]. This is especially true in microsurgery, where models that recognise examples falling outside their reliability boundaries may be safer than models that provide confident classifications every time [35]. In this cohort, uncertainty-abstention led to a subset where senior clinician reinterpretations changed initial risk assessments. Therefore, uncertainty can be viewed as a useful prompt for senior clinician reviews, multidisciplinary discussions, secondary imaging reviews, and verification planning.

Similarly, caution should be exercised when viewing digital twins as described here. Digital twins were not intended as simulators capable of generating alternative scenarios or recommending courses of action. They were weighted case-similarity retrieval systems designed to formalise a common neurosurgical process: comparing a current case to prior cases with similar anatomy, exposure limitations, and reconstruction requirements [36]. Although case-similarity retrieval systems exist in many domains, for extremely rare types of aneurysms, this type of organised retrieval may be helpful. Organised retrieval may help identify prior cases with similar perforator anatomy, relationships to the brainstem, corridor geometry, morphology, planned approach, clip strategy, documented findings, verified rescues, DWI outcomes, and functional results [37].

It is conceptually important that high-distance, low-similarity cases converged with uncertainty abstention. High-distance, low-similarity cases indicate cases with anatomical characteristics unknown to the registry database and may similarly warrant reduced confidence in model predictions. This appears desirable for emerging decision-support platforms, as it directs focus towards cases needing greater human expertise, supportive reviews, and careful planning [38].

### 4.8. Exploratory Relationship to the De Novo BTOCC Construct

As part of this study, BTOCC was created in-house from the same patient population in which it was used as an initial exploratory model of global surgical complexity. It is not an established or externally validated criterion. Therefore, the relationship between BTOCC and each of the other four constructs (PAH-S, BPCI, CCR, and CRB) represents an intra-study comparison among elements derived from the same patient population and does not demonstrate the incremental predictive utility of any individual construct.

Each of the derived parameters represents a separate mechanism-based dimension of complexity related to microsurgery. PAH-S is based upon the hazard posed by perforators, BPCI is based upon brainstem proximity and compression burden, CCR is based upon the constraints imposed by corridors that limit access to structures requiring repair, and CRB reflects the degree to which additional reconstruction burden is introduced during the performance of a complex case. Although these constructs can provide a detailed explanation of why a specific procedure was particularly challenging, the present study does not demonstrate that they have a greater ability to predict difficulty than either BTOCC or any previously established complexity-assessment method. Evaluation outside the current research environment will be necessary to determine whether these mechanism-based constructs provide reliable information beyond that provided by the anatomy and technical characteristics of the procedure.

### 4.9. Clinical Applicability and Translational Meaning

A key aspect of interpreting the results of this project is to view them as a proof-of-concept framework for augmenting microsurgical procedures. While the framework has the potential to enhance structured decision-making processes, it is not designed to replace the expertise of the surgeon. Basilar trunk aneurysm surgery is highly dependent upon the unique characteristics of each patient’s anatomy, the surgeon’s judgement, tactile feedback, visual inspection, flow assessment, and real-time adjustments to the procedure. Therefore, any computational system proposed for use in this setting must be transparent, auditable, and under the control of the surgeon at all times [39].

In addition to these considerations, several clinically relevant applications arise from the score architecture. PAH-S-pre may aid in the identification of high perforator-risk anatomy prior to operation and guide how intensively perforators should be mapped. PAH-S-intra may provide a structured method for documenting exposed anatomy, suspected compromise, and rescue techniques. BPCI may provide a means to identify those patients who need to be counselled about their risks and subsequent follow-up care more sensitively than others based solely on mRS. CCR may facilitate preoperative planning regarding approaches to exposure, anticipated difficulties related to exposure, and clip-applier trajectories. CRB-predictor may aid in clip inventory planning, preparing for possible reconstructive manoeuvres, and planning strategies for verification through angiography. RASP-final may aid in reviewing postoperative technical decisions made during an operation by establishing associations between execution, verification, rescue, and ultimate injury. Case-similarity modules may aid in preparing for conferences and educating residents, fellows, and clinical teams regarding unusual anatomical configurations. Finally, conformal abstention may provide criteria to assist in identifying cases that warrant review by a senior surgeon or multidisciplinary consultation.

As stated above, we have attempted to keep our applications modest and clinically based. We do not envision this as a treatment-selection criterion. Rather, we see its most near-term benefit as providing structured documentation for operations; aiding in educational activities for residents and fellows; assisting in counselling patients regarding their risks; facilitating preoperative planning; providing postoperative surveillance; and assessing quality. These benefits may be of greater relevance in rare aneurysm subtypes where there is little standardisation of experience and very few opportunities to learn from other individuals’ experiences.

Additionally, our goal is to ensure that we maintain an ethically responsible stance regarding our use of this technology. We created this model retrospectively, and no model outputs affected treatment decisions. As a result of our treatment of uncertainty as a safety factor, the potential consequences of relying on model predictions were minimised. For future implementations of similar technologies, we believe that validation outside our centre(s), institutional oversight, data-protection safeguards and applicable privacy regulations, transparent reporting mechanisms, and ongoing monitoring for changes in calibration across institutions will be essential. This planned pathway for implementation aligns with current expectations regarding trustworthiness in the use of clinical artificial intelligence (AI). These expectations include transparency, human oversight, safety, accountability, and ongoing evaluation of performance.

### 4.10. Limitations and Validation Pathway

We view this study as a proof-of-concept analysis focused on a rare and anatomically specific subset of basilar trunk aneurysms that undergo open reconstructive microsurgery. Due to the low incidence of basilar trunk aneurysms requiring open reconstructive microsurgery, the cohort size reflects the rarity of this population. Additionally, due to the consecutive nature of this study and utilisation of a high-resolution registry to document anatomy, operative technique, verification methods utilised in real time, rescue measures taken, and outcome assessments linked together longitudinally, the proposed framework could be evaluated in detail. Our intent was not to create a broadly applicable clinical model but to assess whether microsurgical reasoning could be encoded, temporally separated from outcome information, evaluated internally for adequacy, and explained using language consistent with clinical practice.

While the single-centre design limits broad applicability immediately, it ensured uniformity among the neurosurgical team involved in treating patients with basilar trunk aneurysms using open reconstructive microsurgery; uniformity regarding image review; uniformity in documentation practices; uniformity regarding score creation; and uniformity in adjudicating outcomes during the development phase. Therefore, external validation across multiple independent centres performing neurovascular surgery will be necessary to test transportability across varying surgical environments; varying imaging modalities; various institutional workflows; and varying documentation standards.

Longitudinal intervals between treatments contributed to variability in imaging studies performed during the treatment period; intraoperative verification methods employed; video availability; neuromonitoring trends; documentation density; and perioperative care delivery. However, the longitudinal interval provided us with an opportunity to explore signal stability across changes in technical aspects of the procedure over time. Therefore, future validation should occur prospectively utilising standardised documentation parameters and recalibrating models according to era.

Several scores include expert-adjudicated or semi-quantitative components as well. Expert adjudication and semi-quantitation represent the fact that microsurgical anatomy and operative technique can often not be translated into fully automated measurements. Internally reliable findings were obtained; however, broader reproducibility will require formalised scoring manuals; independent raters; training datasets; and prospective multi-centre testing. Furthermore, although the composite technical/safety failure endpoint appears to represent a singular biological outcome, it should be viewed as an integrated safety construct, as technical safety in basilar trunk aneurysm surgery represents multiple dimensions.

Lastly, all model-performance estimates reported are internal validations of exploratory signals. Consistent with the paradigm described earlier, we chose a conservative strategy for evaluating model performance in this sparse/rare-event scenario. Moreover, evaluative constructs such as RASP-final, PAH-S-post, and CRB-final were not used to predict the same outcomes that they contain. Therefore, the framework should be considered a structured, clinically interpretable foundation for future validation and refinement rather than a deployment-ready decision tool.

### 4.11. Future Directions

The next step involves external validation across additional independent neurovascular centres. Future efforts should test whether PAH-S-pre, BPCI, CCR, CRB-predictor, and RASP-execution retain discrimination; calibration; reliability; explanation stability; and decision-curve utility across differing surgeons; imaging modalities; operative techniques/approaches; and institutional documentation cultures. A multi-centre registry should define standardised terminology and definitions; provide structured scoring guidelines; specify minimum requirements for core imaging studies; annotate video recordings of operative techniques when available; utilise standardised diffusion-weighted imaging postoperatively; assess postoperative angiographic durability; and collect patient-centred outcomes beyond mRS.

Future evaluations should initially focus on assessing feasibility; reliability; incorporation into existing workflows; and calibration before attempting to make direct recommendations concerning treatment options. Initial practical applications may include assistance with preoperative case conference preparation; perforator-risk stratification; verification planning strategies; structured operative documentation techniques; postoperative surveillance plans; morbidity reviews; and educational programmes for residents/fellows regarding risk-adjusted quality metrics [40]. Incorporation into intraoperative decision-making may eventually be feasible depending upon successful integration of intraoperative event logs; ICG interpretations; micro-Doppler waveforms; neuromonitoring trend data; and/or video annotations into existing surgical workflows. The similarity-retrieval module deserves special consideration. Similar prior case retrieval may provide significant value in rare aneurysm subtypes by providing surgeons with enhanced pattern recognition capabilities; improved educational tools for residents/fellows regarding uncommon hazards associated with less commonly encountered anatomical configurations; and improved decision-making abilities by enabling surgeons to anticipate less frequently encountered complications or hazards. If sufficient cases exist within a given institution or network of institutions to justify doing so, federated-validation or privacy-preserving validation methodologies may offer advantages because no single institution is likely to have sufficient numbers of cases to provide robust evidence for broad generalizability [41].

## 5. Conclusions

In general terms, certain aspects of basilar trunk aneurysm surgery may be viewed as temporal, quantifiable, and clinical constructs. Anatomical descriptions of perforators, proximity to the brainstem, limitations of the corridor for clip placement or reconstruction, additional intraoperative rescue manoeuvres, and technical-performance measures allow for the analysis of non-standardised microsurgical techniques at a high level of anatomical detail and specificity.

The cognitive reasoning of clinicians is preserved when computationally relevant models of complex cerebrovascular surgery are constructed. The resultant score values within this consecutive series of patients demonstrated logical and consistent relationships with pontine infarction, angiographically documented occlusion, hidden disability, operative complexity, and composite technical-safety failure. The findings described herein represent an initial stage and therefore require validation using independent datasets. However, these results illustrate how surgeon-based experience may be translated from individual decision-making into collectively comparable and reusable knowledge.

Rather than replacing surgeons’ judgement regarding patient treatment, the proposed model was designed to serve as a resource for surgeons when developing treatment plans. If validated using external datasets, the proposed framework has the potential to improve transparency, accountability, teachability, and measurement in surgical decision-making; facilitate preoperative risk assessment and postoperative monitoring; and support surgical education, training, and quality-of-care assessment.

## Figures and Tables

**Table 1:** Demographic and baseline characteristics with complete count and percentage data. This table summarizes the baseline cohort variables available with complete count-percentage reporting in the final analytical cohort of 31 consecutive patients. It includes cohort size, sex distribution, adult eligibility, and rupture status at presentation. Percentages are calculated using the full cohort as the denominator.

Variable	Category	Count	Percentage (%)
Cohort size	Consecutive patients included	31	100.0
Sex	Female	18	58.1
Sex	Male	13	41.9
Eligibility	Adults aged ≥18 years	31	100.0
Presentation status	Ruptured aneurysm	14	45.2
Presentation status	Unruptured aneurysm	17	54.8

**Table 2:** Operational definitions of the derived microsurgical constructs. The table summarises item-level coding, weights, formulas, temporal eligibility, missing-data handling, and analytical role. Preoperative and intraoperative constructs were outcome-masked, postoperative constructs were restricted to adjudication, and unvalidated thresholds were treated as exploratory.

Construct	Operational Items and Coding	Calculation and Range	Temporal Eligibility and Missing-Data Handling	Interpretation, Analytical Role, and Status
SAPIENS-Dataset: Structured Anatomical and Perforator-Informed Explainable Neurovascular System—Dataset	Six domains, each containing five items: anatomical specificity—segment, morphology, maximum diameter and neck width, projection/orientation, and branch or fenestration anatomy; operative detail—approach/corridor, proximal and distal control, clip construct, verification, and rescue; outcome linkage—early CT/DWI, angiographic outcome, discharge outcome, standardised follow-up outcome, and durability or retreatment; governance—eligibility traceability, source provenance, anonymisation, independent adjudication, and audit trail; documentation completeness—preoperative, intraoperative, postoperative imaging, discharge, and follow-up documentation; and external-validation readiness—data dictionary, reproducible coding, missingness metadata, interoperable export, and scoring manual. Each item: 0 = absent; 1 = incomplete or non-standardised; 2 = complete and standardised; and 3 = complete, standardised, and independently traceable.	Case contribution score = obtained points ÷ maximum eligible points × 100. Maximum raw score when all 30 items are eligible = 90. Dataset-level maturity was summarised using the median and interquartile range of the case contribution scores.	Registry-readiness construct incorporating information from multiple temporal layers. Items that were not applicable were excluded from the eligible denominator; applicable but undocumented items received 0 points.	Higher values indicate greater dataset readiness. Used exclusively to evaluate registry maturity and AI readiness; not entered as a patient-level predictor.
SAPIENS-Model	Six domains scored 0–3. Discrimination: 0, AUC < 0.65; 1, 0.65–0.749; 2, 0.75–0.849; 3, ≥0.85. Calibration: 3, slope 0.90–1.10 and |intercept| ≤ 0.10; 2, slope 0.80–0.89 or 1.11–1.20 and |intercept| ≤ 0.20; 1, slope 0.70–0.79 or 1.21–1.30 or |intercept| 0.21–0.30; 0, poorer or inestimable calibration. Interpretability: 0, unavailable; 1, unstable or ESI < 0.50; 2, ESI 0.50–0.69; 3, ESI ≥ 0.70 with clinically coherent global and local explanations. Decision-curve utility: 0, no positive net benefit; 1, positive at one prespecified threshold; 2, positive at two; 3, positive at three or more. Uncertainty handling: 0, absent; 1, conventional confidence intervals only; 2, bootstrap, Bayesian, or conformal uncertainty; 3, uncertainty estimation plus prespecified abstention. Human oversight: 0, absent; 1, post hoc review; 2, prespecified expert review; 3, expert review with abstention and auditability.	Raw score = sum of six domains, range 0–18. Final score = raw score ÷ 18 × 100.	Calculated only after completion of internal model evaluation. Metrics that could not be estimated received 0 points and were explicitly identified.	Higher values indicate a stronger model-readiness profile. Model-level evaluation construct only; not a patient-level predictor and not evidence of clinical deployability.
BTA-SAPIENS-Case	Ten domains, each containing five items scored 0–2. Anatomy: segment, morphology, size/neck, orientation, branching. Perforators: number, proximity, origin/incorporation, clustering/concealment, territory/confidence. Brainstem/cranial nerves: distance, contact/compression, oedema/mass effect, hydrocephalus, cranial nerve engagement. Corridor: approach, entry point, depth, width, angles/blind spots. Clip reconstruction: count, geometry, construct, parent-vessel remodelling, final configuration. Verification: ICG, micro-Doppler, DSA, endoscopy, neuromonitoring. Rescue: trigger, timing, repositioning, correction, post-rescue verification. Postoperative imaging: CT, DWI, infarct characterisation, vessel patency, residual morphology. Durability: follow-up imaging, follow-up duration, recurrence/growth, migration/stenosis, retreatment. Functional outcome: discharge outcome, longitudinal mRS/GOS, neurological deficits, hidden disability/cognition, work or quality of life. Each item: 0 = not documented; 1 = partially documented; 2 = completely documented.	Final score = obtained points ÷ maximum points among eligible items × 100. Maximum raw score when all 50 items are eligible = 100.	Descriptive construct incorporating multiple temporal layers. Not-applicable items were excluded from the denominator; applicable but undocumented items received 0 points. Missing documentation was not interpreted as absence of a clinical feature.	Higher values indicate greater case-level learning and documentation density. Descriptive only; not used to predict infarction or other clinical outcomes. Associations with treatment era and documentation density were evaluated separately.
PAH-S-pre: Preoperative Perforator-Aware Hazard Score	Visible or suspected perforator number: none, 0; one, 5; two, 10; ≥3, 15. Minimum neck/sac proximity: >5 mm, 0; 1–5 mm, 5; <1 mm or contact, 10. Suspected neck or sac origin: absent, 0; indeterminate, 5; suspected, 10. Clustering: absent, 0; ≥2 clustered perforators, 5. Suspected incorporation: absent, 0; indeterminate, 5; likely, 10. Suspected concealment: absent, 0; possible, 5; likely, 10. Anticipated perforator–clip-line conflict: absent, 0; present, 5. Territory at risk: none identifiable, 0; single focal pontine territory, 3; multiple or bilateral territories, 5. Imaging uncertainty: high confidence, 0; moderate confidence, 5; low confidence, 10.	Raw score = sum of items, range 0–80. Final score = raw score ÷ 80 × 100.	Exclusively preoperative clinical and vascular imaging information available before incision. Confirmed adherence, operative concealment, observed clip-line conflict, dissection difficulty, verification findings, and rescue manoeuvres were excluded. Ratings were outcome-masked and locked before outcome linkage. Unassessable items were coded as unavailable rather than absent.	Higher values indicate greater preoperative perforator hazard. Used for exploratory preoperative risk stratification. PAH-S-pre > 50 was an exploratory anchor point, not a validated treatment threshold.
PAH-S-intra: Intraoperative Perforator-Aware Hazard Score	Directly visualised perforators: none, 0; 1–2, 5; ≥3, 10. Operatively concealed perforators: absent, 0; suspected, 5; confirmed, 10. Confirmed origin/adherence: absent, 0; one feature, 5; both or multiple sites, 10. Perforator–clip-line conflict: absent, 0; suspected, 5; directly observed, 10. Dissection difficulty: routine, 0; moderate, 2; severe, 5. Temporary occlusion: none, 0; <180 s, 1; 180–300 s, 2; 300–600 s, 4; >600 s, 5. Suspected compromise: absent, 0; transient/equivocal, 5; persistent or confirmed, 10. Repositioning/corrective action: none, 0; one correction, 5; ≥2 corrections or major rescue, 10. Abnormal verification finding: ICG, micro-Doppler, neuromonitoring, and intraoperative angiography each scored 0 if normal and 5 if abnormal.	Raw score = sum of eligible items; maximum = 90. Final score = raw score ÷ maximum eligible points × 100.	Operative information available after exposure and before postoperative imaging. Raters remained masked to postoperative DWI, angiographic outcomes, functional outcomes, and mortality. A verification modality that was not performed was coded as unavailable and was not treated as normal.	Higher values indicate greater updated intraoperative perforator hazard. Used for pre-outcome intraoperative risk updating and rescue assessment; not a postoperative adjudication construct.
PAH-S-post: Postoperative Perforator-Aware Hazard Score	DWI injury burden: absent, 0; focal/punctate, 10; territorial or multifocal, 20. Pontine involvement: absent, 0; unilateral/focal, 10; bilateral or extensive, 20. Angiographic compromise: absent, 0; stenosis, 10; occlusion, 20. New anatomically concordant brainstem deficit: absent, 0; transient or mild, 10; persistent or major, 20. Mechanistic attribution: unlikely, 0; possible, 10; probable or definite, 20.	Raw and final score = sum of five items, range 0–100.	Postoperative imaging, angiographic, and clinical information only. Missing postoperative imaging was coded as unavailable and did not imply absence of injury.	Higher values indicate greater adjudicated perforator-related injury burden. Used exclusively for mechanistic adjudication and never to predict any component incorporated into the score.
RASP-execution: Rescue-Adjusted Surgical Precision—Execution	Proximal control: inadequate, 0; limited/delayed, 3; secure, 5. Distal control: same coding. Clip-reconstruction execution: unsafe/incomplete, 0; adequate after revision, 5; stable final construct, 10. Perforator preservation: unresolved compromise, 0; uncertain/partially preserved, 5; preserved by direct or adjunct verification, 10. Repositioning: required but ineffective, 0; effective after multiple attempts, 3; not required or effective after one correction, 5. Verification response: abnormal and unresolved, 0; equivocal, 3; normal or corrected, 5. Neuromonitoring response: persistent deterioration, 0; partial recovery, 3; stable or complete recovery, 5. Correction of suspected compromise: absent despite indication, 0; partial, 3; complete, 5.	Starting value = 50. Performance contribution = obtained item points ÷ maximum eligible item points × 50. RASP-execution = 50 + performance contribution; theoretical range 50–100.	Intraoperative information available before postoperative imaging or clinical outcome disclosure. Not-applicable rescue items and unavailable optional modalities were excluded from the eligible denominator and were not treated as successful performance.	Higher values indicate safer intraoperative execution and rescue. Pre-outcome technical-performance construct only.
RASP-final: Rescue-Adjusted Surgical Precision—Final	RASP-final = RASP-execution − 8 for pontine infarction −5 for residual neck or −10 for residual sac −8 for retreatment −20 for death attributable to a surgical complication. Residual-neck and residual-sac penalties were mutually exclusive.	RASP-final = max [0, RASP-execution − cumulative penalties]. Range 0–100.	Postoperative technical-performance endpoint requiring imaging and clinical outcome information.	Higher values indicate safer final technical performance. Not used as an independent predictor of postoperative injury because postoperative harm is incorporated into its calculation.
BPCI: Brainstem Proximity–Compression Index	Let Psac = max [0, min(1, (5 − sac-to-pons distance in mm)/5)]; Pneck = analogous neck-to-pons proximity; C = 0 for no contact, 0.5 for contact, and 1 for compression; G = compression grade/3; E = pontine oedema, 0/1; M = midbrain or ventricular compression, 0/1; H = hydrocephalus, 0/1; and CN = sum of cranial nerve contact grades divided by the maximum possible score among assessed nerves.	BPCI = 0.15Psac + 0.10Pneck + 0.15C + 0.20G + 0.10E + 0.10M + 0.05H + 0.15CN. Range 0–1.	Preoperative imaging only. Missing MRI-dependent components were coded as unavailable, not absent. The index was calculated when at least six of eight components were available; weights were proportionally renormalised across available components.	Higher values indicate greater brainstem proximity–compression burden. Used for exploratory analyses of brainstem vulnerability, functional outcome, and hidden disability.
CCR-planned: Planned Corridor Constraint Ratio	D = distance in millimetres from the centre of the planned dural corridor entry point to the centre of the aneurysm neck along the planned operative trajectory. W = minimum corridor diameter perpendicular to that trajectory at the point of maximal anatomical constraint, measured before operative exposure or retraction. Measurements were made using the three-dimensional measurement tool in Brainlab Elements. Three measurements were obtained per case and averaged.	CCR-planned = D/W. Continuous ratio with no fixed upper bound.	Derived exclusively from preoperative approach simulation. Intraoperatively achieved corridor width was recorded separately as CCR-achieved and was not substituted into the preoperative predictor. In the reliability subset, measurements were repeated independently by both raters.	Higher values indicate a deeper and narrower planned corridor. CCR was interpreted as a combined anatomical and approach-dependent descriptor. Because corridor width depends partly on approach selection and planned skull-base exposure, associations with operative duration and complexity were considered partly structural and not causal. CCR > 1.5 was an exploratory anchor point.
CRB-predictor: Clip Reconstruction Burden—Pre-outcome	Applied clip count: one, 0; two, 5; three, 10; ≥4, 15. Most complex clip geometry: straight, 0; curved, 3; angled, 5; fenestrated, 10; only the highest category was counted. Tandem or stacked construct: absent, 0; present, 8. Parent-vessel reconstruction: absent, 0; present, 10. Dome/neck remodelling: absent, 0; present, 5. Highest-level adjunct strategy: none, 0; wrapping, 5; trapping, 15; bypass-assisted reconstruction, 20; categories were mutually exclusive. Clip slippage: absent, 0; present, 5. Repositioning: none, 0; one, 3; two, 6; ≥3, 10.	CRB-predictor = sum of applicable points, range 0–83.	Pre-outcome intraoperative construct based on the applied clip configuration and corrective actions completed before postoperative imaging. Missing operative documentation was coded as unavailable rather than 0.	Higher values indicate greater reconstruction burden. Used exploratorily in analyses of operative burden and angiographic outcome. It is not a purely preoperative predictor.
CRB-final: Clip Reconstruction Burden—Final	CRB-final = CRB-predictor + postoperative residual morphology: no residual, 0; residual neck, 5; residual sac, 10.	Range 0–93.	Postoperative descriptive endpoint requiring final angiographic information.	Higher values indicate greater final reconstruction complexity. Used only for technical description and outcome interpretation; not used to predict occlusion because residual morphology is incorporated into the score.
BTOCC total/class: Basilar Trunk Operative Complexity Classification	Seven domains, each scored 0–4. Perforator burden: 0, none adjacent; 1, one adjacent without incorporation; 2, multiple or clustered; 3, suspected/confirmed incorporation or concealment; 4, multiple incorporated perforators or territories. Brainstem hazard: 0, >5 mm without contact; 1, 3–5 mm; 2, <3 mm or contact; 3, compression or oedema; 4, major distortion or hydrocephalus. Corridor difficulty: 0, direct and unrestricted; 1, mildly restricted; 2, moderate restriction requiring angle adjustment; 3, severe restriction requiring additional exposure or mobilisation; 4, persistent blind spots or multiple corridor changes. Clip reconstruction: 0, single standard clip; 1, two-clip simple construct; 2, multi-clip or fenestrated construct; 3, tandem/stacked or parent-vessel reconstruction; 4, trapping, bypass, or repeated major revision. Proximal-control difficulty: 0, direct and secure; 1, mildly restricted; 2, temporary or adjunct control required; 3, remote, difficult, or unstable control; 4, complex flow-arrest or bypass-dependent control. Cranial nerve crowding: 0, none; 1, contact without restriction; 2, one nerve limiting the corridor; 3, multiple nerves requiring manipulation; 4, severe crowding with major injury risk. Aneurysm-wall complexity: 0, regular and pliable saccular wall; 1, focal irregularity, thrombus, or calcification; 2, broad neck or partial thrombosis; 3, fusiform, dissecting, giant, or extensively calcified anatomy; 4, anatomy requiring segmental reconstruction or trapping.	BTOCC total = sum of seven components, range 0–28. Class I = 0–7; Class II = 8–14; Class III = 15–21; Class IV = 22–28.	Pre-outcome complexity construct combining preoperative and intraoperative information available before postoperative imaging. Missing components prevented class assignment unless at least six of seven domains were available; when six were available, the total was rescaled to a 28-point equivalent before classification.	Higher values indicate greater operative complexity. Developed de novo in the present cohort and used as an exploratory internal comparator. It is not a previously published or externally validated reference standard. Comparisons against BTOCC were interpreted as exploratory internal comparisons, not evidence of incremental value over an established benchmark.

**Table 3:** Summary of score distributions, exploratory thresholds, target endpoints, and principal univariate signals for the derived microsurgical intelligence and complexity scores. Values are reported as median and interquartile range unless otherwise specified. AI, artificial intelligence; AUC, area under the receiver operating characteristic curve; BPCI, Brainstem Proximity–Compression Index; BTA, basilar trunk aneurysm; BTA-SAPIENS-Case, case-level Structured Anatomical and Perforator-Informed Explainable Neurovascular System score; BTOCC, Basilar Trunk Operative Complexity Classification; CCR, corridor constraint ratio; CI, confidence interval; CRB, clip reconstruction burden; IQR, interquartile range; LOOCV, leave-one-out cross-validation; OR, odds ratio; PAH-S, perforator-aware hazard score; RASP, rescue-adjusted surgical precision; SAPIENS, Structured Anatomical and Perforator-Informed Explainable Neurovascular System; ρ, Spearman’s rank correlation coefficient.

Score	Distribution/Threshold	Main Endpoint or Comparator	Principal Signal
SAPIENS-Dataset	Median, 74; IQR, 68–79; range, 58–86; AI-development-ready, 18/31 (58.1%); explainable-AI prototype, 7/31 (22.6%)	Dataset maturity	25/31 cases (80.6%) reached AI-development-ready or prototype-level status
BTA-SAPIENS-Case	Median, 78; IQR, 71–84	Case-level documentation and learning density	Descriptive construct only; associations with treatment era, imaging availability, and documentation intensity were evaluated separately
PAH-S-pre total	Median, 48; IQR, 39–57; range, 22–79; PAH-S > 50 in 13/31 (41.9%)	New postoperative pontine infarction	OR, 5.7; 95% CI, 1.3–25.1; *p* = 0.02; LOOCV AUC, 0.85; 95% CI, 0.71–0.96
RASP-final	Median, 81; IQR, 73–88	Technical-performance endpoint	Median 84 without pontine infarction vs. 72 with infarction; *p* = 0.003
BPCI	Median, 0.37; IQR, 0.22–0.66; BPCI > 0.5 in 12/31 (38.7%)	Brainstem syndrome; hidden disability	Brainstem syndrome: OR, 6.8; 95% CI, 1.4–33.1. Hidden disability: OR, 7.2; 95% CI, 1.5–35.8
CCR	Median, 1.31; IQR, 1.02–1.72; CCR > 1.5 used as high-constraint anchor	Operative time; clip repositioning; surgeon-rated complexity	Operative time: ρ = 0.61, *p* = 0.001. Clip repositioning: ρ = 0.55, *p* = 0.002. Surgeon-rated complexity: ρ = 0.64, *p* < 0.001
CRB-predictor	Median, 20; IQR, 13–31	Complete occlusion; operative time	Complete occlusion: OR per 5 points, 0.85; 95% CI, 0.73–0.97. Operative time: ρ = 0.69, *p* < 0.001
BTOCC total	Median, 8; IQR, 6–11	RASP-final; complete occlusion	RASP-final: ρ = −0.64, *p* < 0.001. Complete occlusion: OR per point, 0.68; 95% CI, 0.48–0.96

**Table 4:** Internal validation and benchmarking of the composite technical-safety failure model. The table summarises discrimination, calibration, optimism correction, nonlinear model comparison, and rule-based interpretability, including the confusion matrix and performance metrics for the exploratory CART-derived risk phenotype.

Domain	Model/Method	Metric	Result
Primary model	Firth penalised logistic regression	Predictors	PAH-S-pre + BPCI + CCR + CRB-predictor + rupture status
Internal validation	Primary Firth model	LOOCV folds	31
Optimism correction	Primary Firth model	Bootstrap replicates	2000
Discrimination	Primary Firth model	LOOCV ROC AUC	0.91; 95% CI, 0.83–0.97
Optimism-adjusted discrimination	Primary Firth model	Corrected ROC AUC	0.88; 95% CI, 0.79–0.95
Classification	Primary Firth model	Balanced accuracy/F1/MCC	0.84/0.79/0.68
Calibration	Primary Firth model	Intercept/slope/Brier/ICI	–0.08/0.96/0.10/0.045
Null testing	Permutation analysis	*p*-value	<0.001
Nonlinear benchmark	Random forest/monotonic XGBoost	ROC AUC range	0.89–0.90
Nonlinear calibration	Random forest/monotonic XGBoost	Calibration slope/Brier	0.85–0.86/0.13
EBM signal	Explainable Boosting Machine	PAH-S and CCR behaviour	PAH-S ≈ 40; CCR > 1.5
Rule extraction	Shallow classification tree	PAH-S > 53 and CCR > 1.6	Failure in 5/6 rule-positive cases; estimated risk 83%
Rule-negative stratum	Shallow classification tree	Rule absent	Failure in 3/25 cases; estimated risk 11%
Rule-based confusion matrix	Shallow classification tree	TP/FP/FN/TN	5/1/3/22
Rule-based sensitivity and specificity	Shallow classification tree	Sensitivity/specificity	62.5%/95.7%
Rule-based predictive values	Shallow classification tree	PPV/NPV	83.3%/88.0%
Rule-based summary metrics	Shallow classification tree	Balanced accuracy/F1/MCC	0.79/0.71/0.64

## Data Availability

Available upon reasonable request from the corresponding author.
